# Dudomycins: New Secondary Metabolites Produced after Heterologous Expression of an Nrps Cluster from *Streptomyces albus* ssp. *Chlorinus* Nrrl B-24108

**DOI:** 10.3390/microorganisms8111800

**Published:** 2020-11-16

**Authors:** Constanze Lasch, Marc Stierhof, Marta Rodríguez Estévez, Maksym Myronovskyi, Josef Zapp, Andriy Luzhetskyy

**Affiliations:** 1 Department of Pharmaceutical Biotechnology, Saarland University, 66123 Saarbruecken, Germany; constanze.lasch@uni-saarland.de (C.L.); m.stierhof@t-online.de (M.S.); marta.rodriguezestevez@uni-saarland.de (M.R.E.); maksym.myronovskyi@uni-saarland.de (M.M.); 2 Department of Pharmaceutical Biology, Saarland University, 66123 Saarbruecken, Germany; j.zapp@mx.uni-saarland.de; 3 AMEG Department, Helmholtz Institute for Pharmaceutical Research Saarland, 66123 Saarbruecken, Germany

**Keywords:** Streptomyces, NRPS, heterologous expression

## Abstract

Since the 1950s, natural products of bacterial origin were systematically developed to be used as drugs with a wide range of medical applications. The available treatment options for many diseases are still not satisfying, wherefore, the discovery of new structures has not lost any of its importance. Beyond the great variety of already isolated and characterized metabolites, Streptomycetes still harbor uninvestigated gene clusters whose products can be accessed using heterologous expression in host organisms. This works presents the discovery of a set of structurally novel secondary metabolites, dudomycins A to D, through the expression of a cryptic NRPS cluster from *Streptomyces albus* ssp. *Chlorinus* NRRL B-24108 in the heterologous host strain *Streptomyces albus* Del14. A minimal set of genes, required for the production of dudomycins, was defined through gene inactivation experiments. This paper also proposes a model for dudomycin biosynthesis.

## 1. Introduction

Biologically active natural products of microbial origin are the result of natural design and evolutionary optimization to target essential biological processes, and are, therefore, a valuable source of potential drug leads [1,2,3]. Since the 1950s, the bacterial genus of Streptomyces largely contributed to the pool of diverse structural scaffolds, some of which were developed as successful drugs, e.g., vancomycin (antibacterial) [4], avermectin (antiparasitic) [5,6] and actinomycin D (anticancer) [7,8], etc. As a result of extensive screening, the discovery of structurally novel compounds with new biological targets has become a challenging task nowadays. However, only a small part of the biosynthetic gene clusters encoded in microbial genomes is readily expressed in the laboratory, while the rest remain silent. It seems that under standard conditions, only a confined number of clusters leads to the production of natural products, which are then often rediscovered. Identification of unique biosynthetic pathways within genome sequence data and their targeted expression in optimized chassis strains is regarded as a most promising approach to access new biologically active scaffolds [9,10].

Recently we have reported the first studies on the genome mining of the strain *Streptomyces albus* ssp. *Chlorinus* NRRL B-24108. Heterologous expression of *S. albus* ssp. *Chlorinus* genes enabled identifying the biosynthetic gene clusters of the antibiotic nybomycin and the herbicide albucidin, as well as the isolation and characterization of the novel compounds benzanthric acid and fredericamycin C2 [11,12,13,14]. This study reports on a biosynthetic gene cluster of *Streptomyces albus* ssp. *Chlorinus* encoding an uncharacterized nonribosomal peptide synthetase. The bioinformatic analysis did not reveal any characterized homologs of the studied cluster, implying that it might encode a novel natural product. Heterologous expression of the NRPS cluster in our optimized hosts *Streptomyces albus* Del14 [15] and *Streptomyces lividans* Del8 [16] led to identifying a set of four new compounds we named dudomycins. The compounds were purified, and their structures were elucidated in ^1^H and ^13^C nuclear magnetic resonance (NMR) experiments. The identified compounds are structurally related and consist of a core lysine and three branched-chain hydroxy fatty acid residues. A hypothesis on dudomycin biosynthesis is proposed based on the results of gene deletion experiments.

## 2. Materials and Methods

### 2.1. General Experimental Procedures

The strains, bacterial artificial chromosomes (BACs), and plasmids used in this work are listed in Table S3. *Escherichia coli* strains were cultured in lysogeny broth (LB) medium [17]. Streptomyces strains were grown on soy flour mannitol (MS) agar [18] and in liquid tryptic soy broth (TSB; Sigma-Aldrich, St. Louis, MO, USA). Liquid DNPM medium (40 g/L dextrin, 7.5 g/L soytone, 5 g/L baking yeast, and 21 g/L MOPS, pH 6.8 as aqueous solution) and defined medium DM (mannitol 5 g/L, amino acid 0.5 g/L, K2PO4 0.5 g/L, MgSO4 × 7 H2O 0.2 g/L, FeSO4 × 7 H2O 0.01 g/L) were used for metabolite expression. When DM was used for production, the cells of the preculture were washed three times using amino acid-free defined medium prior to inoculation. Amino acids l-val, l-ile, l-lys, and d-lys were supplied to a defined medium as needed. The antibiotics kanamycin, apramycin, hygromycin, ampicillin, and nalidixic acid were added when required.

### 2.2. Isolation and Manipulation of DNA

DNA manipulation, transformation into *E. coli*, as well as intergeneric conjugation between *E. coli* and Streptomyces, were performed according to standard protocols [17,18,19]. BAC DNA from a constructed genomic library of *Streptomyces albus* ssp. *Chlorinus* NRRL B-24108 was isolated with the BACMAX™ DNA purification kit (Lucigen, Middleton, WI, USA). Deletion of several genes was performed on the BAC 4E8 itself using a two-step approach. Step one focused on deleting all genes 1 to 21, leading to BAC 4E8_del1, step two addressed further deletion of genes 25 and 26 on BAC 4E8_del1, resulting in BAC 4E8_del2. The genes were replaced by resistance markers ampicillin and hygromycin through homologous recombination using the Red/ET system [20]. PCR was performed for amplification of the respective gene cassettes from plasmids pUC19 and pXCM hygformax. PCR primers 20190429_1_fw, 20190429_1_rev, 20190429_2_fw, and 20190429_2_rev were constructed with overhang regions for site-specific introduction of the cassettes left or right from the dudomycin cluster and simultaneous removal of the genes 1 to 21 or 25 and 26. Restriction mapping and sequencing were used to control the success of the recombination. Restriction enzymes from ThermoFisher Scientific (Waltham, MA, USA) or New England BioLabs NEB (Ipswich, MA, USA) were used according to the manufacturer’s instruction.

### 2.3. Metabolite Extraction

For metabolite extraction, Streptomyces strains were grown in 15 mL of TSB in a 100 mL baffled flask for 1 to 2 days, and 1 mL of seed culture was used to inoculate 100 mL of production medium in a 500 mL baffled flask. Cultures were grown for 7 days at 28 °C and 180 rpm in an Infors multitron shaker (Infors AG, Basel, Switzerland). Metabolites were extracted from the culture supernatant with an equal amount of either *n-*butanol or ethyl acetate, evaporated at 40 °C and kept at storage condition 4 °C.

### 2.4. Mass Spectrometry (MS) Metabolite Analysis

Dried extracts were dissolved in methanol prior to the mass spectrometry (MS) analysis. MS experiments were carried out on a Dionex Ultimate 3000 UPLC system (ThermoFisher Scientific, Waltham, MA, USA) coupled to PDA detector (stationary phase 30 or 100 mm ACQUITY UPLC BEH C18 1.7 µm column (Waters Corporation, Milford, MA, USA), mobile phase: Linear gradient of [A] ddH_2_O + 0.1% formic acid/[B] acetonitrile + 0.1% formic acid, 5% to 95% at flow rate of 0.6 mL/min). Further mass detection was performed coupling either an amaZon speed (Bruker, Billerica, MA, USA) or LTQ Orbitrap XL mass spectrometer (ThermoFisher Scientific, Waltham, MA, USA) using positive ionization mode and mass range detection of m/z 200 to 2000. Data analysis was performed using software Compass Data Analysis v. 4.1 (Bruker) and Xcalibur v. 3.0 (ThermoFisher Scientific).

### 2.5. Purification

The extract from the 10 L culture was dissolved in methanol. A first purification step was carried out using Size Exclusion Chromatography (SEC; stationary phase: Sephadex-LH20; mobile phase: isocratic elution using 100% methanol). Fractions containing dudomycins were pooled, dried, redissolved in methanol and undergone a second purification step: Reversed Phase (RP) HPLC (Agilent Infinity 1200 series HPLC system; stationary phase: Synergi^TM^ 4 µm Fusion-RP 80 Å 250 × 10 (Phenomenex, Torrance, CA, USA); mobile phase: Linear gradient of [A] H_2_O + 0.1% formic acid/[B] acetonitrile + 0.1% formic acid, 30% to 95% [B] in 14.5 min at flow rate of 4 mL/min, column oven temperature 45 °C, detection UV 210 nm followed by fraction control on HPLC-MS). Fractions were pooled to obtain the four pure dudomycin isolates A to D.

### 2.6. Nuclear Magnetic Resonance (NMR) Spectroscopy

The ^1^H-NMR spectrum in CDCl_3_ (Deutero, Kastellaun, Germany) was recorded on a Bruker Avance 500 spectrometer (Bruker, BioSpin GmbH, Rheinstetten, Germany) equipped with a 5 mm BBO probe at 298 K. The chemical shifts were reported in parts per million (ppm) relative to TMS. The spectra were recorded with the standard ^1^H pulse program using 64 scans. All other NMR spectra were acquired on a Bruker Ascend 700 MHz NMR spectrometer at 298 K equipped with a 5 mm TXI cryoprobe. As a solvent, deuterated CD_3_OD was used. HSQC, HMBC, 1H-1H COSY spectra were recorded using the standard pulse programs from the TOPSPIN v. 3.6 software. Selective 1D TOCSY experiments were performed using mixing times of 120 ms.

### 2.7. Genome Mining and Bioinformatic Analysis

The genome of *S. albus* ssp. *Chlorinus* was screened for secondary metabolite biosynthetic gene clusters using the antiSMASH online tool (https://antismash.secondarymetabolites.org/#!/start) [21]. Analysis of genetic data was performed using Geneious software, v. 11.0.3 [22]. The genomic sequence of *Streptomyces albus* ssp. *Chlorinus* was deposited in GenBank under accession number VJOK00000000. For dereplication, the Dictionary of Natural Products (DNP) 28.1 was used as a database of known natural products.

## 3. Results and Discussion

### 3.1. Identification and Expression of the NRPS Gene Cluster

**Figure 1:**
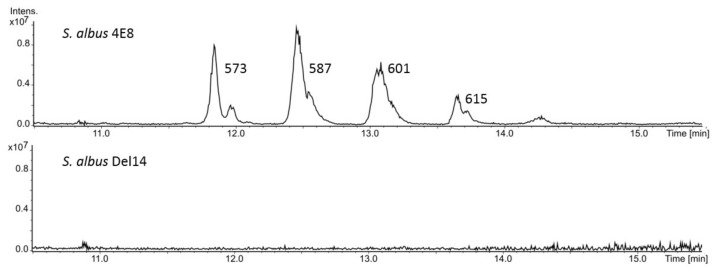
HPLC-MS analysis of dudomycin production by *S. albus* Del14 strain harboring the BAC (bacterial artificial chromosomes) 4E8. The strain *S. albus* Del14 without the BAC was used as a control. The extracted base peak chromatograms 573-574 Da, 587-588 Da, 601-602 Da, 615-616 Da are shown.

Genome mining of *Streptomyces albus* ssp. *Chlorinus* NRRL B-24108 using AntiSMASH software revealed several cryptic biosynthetic gene clusters (BGCs) within its chromosome [22]. A BGC encoding a putative nonribosomal peptide synthetase (NRPS) caught our attention as the software did not detect any homology to already characterized BGCs [23]. A BAC 4E8 covering the entire NRPS cluster was identified in the previously constructed genomic library of *S. albus* ssp. *Chlorinus* (GenBank accession number VJOK00000000). The BAC 4E8 was transferred into the optimized heterologous host strains *Streptomyces albus* Del14 and *Streptomyces lividans* Del8, leading to the respective exconjugant strains *Streptomyces albus* 4E8 and *Streptomyces lividans* 4E8. The obtained exconjugant strains were cultivated in the production medium DNPM, and the metabolites were extracted from the culture supernatant with ethyl acetate or *N-*butanol. High-resolution HPLC-MS analysis revealed the presence of four new peaks in the ethyl acetate and *N*-butanol extracts of both *S. albus* and *S. lividans* harboring the BAC 4E8 (Figure 1; Figures S1 and S2). The identified peaks could not be observed in the extracts of the control strains without the BAC. Analysis of the mass spectra of the identified peaks revealed the molecular ions [M + H^+^] with the masses 573.411, 587.426, 601.442, and 615.457 Da. A search in the DNP database of natural products for the identified high-resolution masses did not generate any matches, implying that the identified compounds might be new. The mass differences of 14 Da between the individual compounds imply that they might differ by the presence of additional CH_2_ groups. The identified compounds were named dudomycins A, B, C, and D.

### 3.2. Purification and Structure Elucidation

To get insights into the structures of the produced compounds, the producer strain *S. albus* 4E8 was cultivated in 10 L of the production medium DNPM, and the metabolites were extracted from the culture supernatant with ethyl acetate. The strain *S. albus* 4E8 was preferred to *S. lividans* 4E8, due to its higher production level. The dudomycins A to D corresponding to the identified molecular ions [M + H^+^] with the masses 573.411, 587.426, 601.442, and 615.457 Da were successfully purified from the extract: 1.2 mg of dudomycin A, 1.1 mg of dudomycin B, 0.7 mg of dudomycin C and 0.5 mg of dudomycin D were obtained. The structure elucidation of the isolated dudomycins was performed using 1D and 2D NMR with a special focus on 1D TOCSY experiments.

Due to its small molecular mass, dudomycin A was the first compound used for the structure elucidation. The molecular formula was calculated as C_30_H_56_O_8_N_2_ based on the calculated high-resolution mass of 572.403 Da, indicating four degrees of unsaturation. Analysis of ^1^H, ^13^C NMR, and edited HSQC led to 6 methyls, 13 methylenes, 7 methines, and 4 quaternary carbons. Two of the remaining five protons belonged to NH-groups, as the ^1^H NMR measurement in CDCl_3_ revealed the presence of two signals at δH 6.81 and δH 7.12 ppm (Figure S3), which disappeared in protic solvents. Due to broad signals and the compound’s poor solubility in CDCl_3_, all other spectra were recorded in CD_3_OD.

Interpretation of HHCOSY revealed four discrete spin systems in the molecule. The first sequence starting from the methine signal at δH 4.26 followed by four methylenes at δH 1.83, 1.38, 1.51, and 3.17 was assigned to lysine, which was supported by HMBC data. The remaining three spin systems were mostly close to each other, leading to many overlaps of methylene and methyl resonances in the area of δH 0.8–1.7 (Figure S4). However, well-separated methine proton signals at δH 5.20, 3.95, 3.92 enabled selective 1D TOCSY experiments using these resonances as irradiation points. Their careful interpretation enabled the full assignment of all spin systems leading to three different 3-hydroxy-6-methyl heptanoic acid moieties (HMH1 to HMH3).

**Figure 2:**
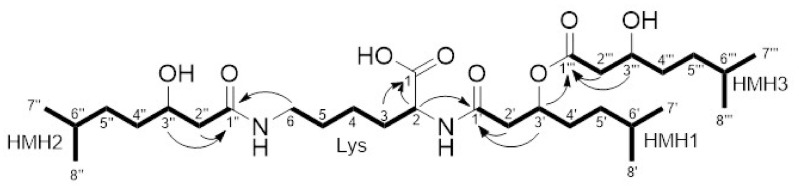
Observed HMBC (arrows) and selective 1D TOCSY (bold lines) key correlations of dudomycin A.

Long-range HMBC correlations of Lys-H-2 (δH 4.26, δC 56.2) to HMH1-C-1′ (δC 171.9) and Lys-H-6 (δH 3.17, δC 40.4) to C-1′ (δC 174.3) proved connections of HMH1 and HMH2 via amide bonds. The carbonyl group of lysine (δC 178.9) did not show any external connections, hence it was assigned as a free carboxyl group. The remaining long-range HMBC correlations between H-3′ (δH 5.20, δC 73.3) and C-1‴ (δC 173.2) indicated an ester bond between HMH1 and HMH3 leading to the final structure of dudomycin A (Figure 2; Figures S3–S10; Table S1).

The results of NMR analysis indicate that the dudomycins B (C_31_H_58_O_8_N_2_), C (C_32_H_60_O_8_N_2_), and D (C_33_H_62_O_8_N_2_) are structurally related to dudomycin A. From these compounds, only dudomycin D (Table S2) was a pure substance, while dudomycins B and C were the mixtures of several isomers. Similar to dudomycin A, the structure of dudomycin D contains a lysine core, with three hydroxy fatty acids attached. However, in contrast to dudomycin, A three residues of 3-hydroxy-6-methyl octanoic acid (HMO1 to HMO3) can be found in the structure of dudomycin D instead of three residues of 3-hydroxy-6-methyl heptanoic acid (HMH1 to HMH3). The alkyl chain of the 3-hydroxy-6-methyl octanoic acid is extended by one additional CH_2_ group compared to 3-hydroxy-6-methyl heptanoic acid what explains the overall mass difference of 42 Da between dudomycins A and D.

**Figure 3:**
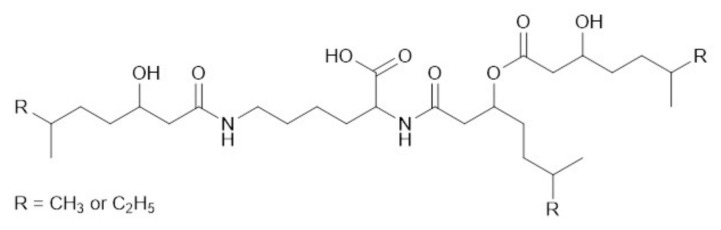
The structures of isolated dudomycins. In the case of dudomycin A, all three R groups correspond to the CH_3_ group. Dudomycin B is a mixture of 3 constitutional isomers with two R groups corresponding to CH_3_ and one R group—to C_2_H_5_. Dudomycin C is a mixture of 3 constitutional isomers with one R group corresponding to CH_3_ and two R groups—to C_2_H_5_. In dudomycin D, all three R groups correspond to C_2_H_5_.

NMR spectra of dudomycins B and C revealed that both samples consist of three isomers each. Similar to dudomycins A and D, dudomycins B and C also contain a lysine core in their structures. In contrast to dudomycins A and D, which contain either HMH or HMO residues bound to the core, dudomycins B and C contain a mixture of HMH and HMO residues in their structures. The results of ^1^H NMR and a multitude of 1D TOCSY measurements indicate that dudomycin B contains two HMH and one HMO residues, while dudomycin C contains one HMH and two HMO residues. These results are in accordance with the observed mass difference of 14 Da between Dudomycin A and B and of 28 Da between dudomycins A and C. Three isomeric forms are possible for both dudomycin B and C (Figure 3, Figures S11–S24).

Experiments to determine the absolute configuration of dudomycins have not been performed, due to low amounts of the isolated compounds. The results of a feeding experiment imply that l-lysine is used as a precursor for dudomycin biosynthesis (Figure S25). A higher dudomycin production level was observed upon the cultivation of *S. albus* 4E8 in a defined medium with l-lysine as a nitrogen source than in the medium with d-lysine.

The occurrence of HMH and HMO in natural products is very rare, and to the best of our knowledge, the structures of dudomycin A to D have not been reported before.

### 3.3. Determination of the Minimal Dudomycin Gene Cluster

**Figure 4:**
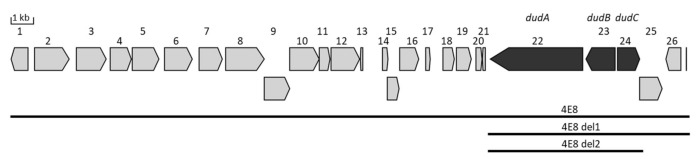
The chromosomal fragment of *Streptomyces albus* ssp. *Chlorinus* NRRL B-24108 containing the dudomycin gene cluster. The genes putatively involved in dudomycin biosynthesis are highlighted in dark grey. The chromosomal fragments cloned in BACs 4E8, 4E8_del1 and 4E8_del2 are shown with black bars.

The 30 kb DNA fragment cloned in the BAC 4E8 contains 26 genes (Figure 4; Table 1). Gene 22 encodes a putative NRPS comprising condensation (C), adenylation (A), peptidyl carrier protein (PCP), and thioesterase (TE) domains. The A domain was predicted to have a weak preference for recognition of the amino acid ornithine. Due to the structural similarity of ornithine and lysine gene 22 encoding, an NRPS enzyme was regarded to be involved in dudomycin biosynthesis. A sequence analysis of the regions upstream and downstream of gene 22 was performed to identify the genes possibly involved in the production of dudomycins. This analysis did not reveal any gene whose product could be enzymatically involved in dudomycin biosynthesis. Sequence homology analysis revealed that the homologs of genes 22, 23, and 24 are clustered together in the genomes of seven different strains implying that those genes might be involved in the same pathway. Genes 23 and 24 encode a putative transport protein and a putative transcriptional regulator, respectively. To confirm that the genes upstream, gene 22 are not involved in dudomycin biosynthesis, the genes 1 to 21 were substituted in the BAC 4E8 with an ampicillin cassette using Red/ET. The constructed BAC 4E8_del1 was transferred into *S. albus* Del14 strain yielding *S. albus* 4E8_del1. HPLC-MS analysis of the metabolite production by the obtained strains did not detect any differences in dudomycin production compared to the *S. albus* 4E8 strain. This clearly indicated that the genes 1 to 21 encoded in the BAC 4E8 are not involved in the biosynthesis of dudomycins.

To find out if genes 25 and 26 are involved in dudomycin production, they were replaced in the BAC 4E8_del1 with a hygromycin resistance cassette using Red/ET. The constructed BAC 4E8_del2 contains genes 22, 23, and 24 only. The BAC was transferred into *S. albus* Del14, and the obtained strain *S. albus* 4E8_del2 was checked for dudomycin production. HPLC-MS analysis revealed that deleting genes 25 and 26 did not affect the production of dudomycins (Figure S26), indicating that these genes do not take part in the biosynthesis of the compounds.

The results of gene deletion experiments demonstrate that genes 22, 23, and 24 (designated as *dudA*, *dudB*, and *dudC*), encoding the putative NRPS, transport protein, and transcriptional regulator (Table 1, Figure 4), suffice for the production of dudomycins. Since the products of *dudB* and *dudC* do not have an enzymatic function, we suppose that only the product of *dudA* is responsible for the biosynthesis of dudomycins. The products of *dudB* and *dudC* are likely involved in the transport of the biosynthetic products and in the regulation of dudomycin production, respectively. Since the inactivation of the genes *dudB* and *dudC* was not performed, the possibility that the genes are not essential for dudomycin production cannot be completely excluded.

### 3.4. Biosynthesis of Dudomycins

**Figure 5:**
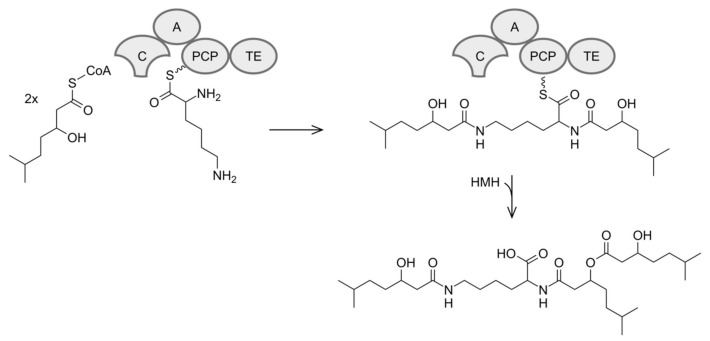
Proposed biosynthesis exemplarily for dudomycin A.

Structurally dudomycins consist of a lysine core and three hydroxy fatty acid residues, two of which are attached directly to the lysine moiety through amide bonds, while the third residue is esterified with one of the lysine bound hydroxy fatty acids (Figure 3). Two types of hydroxy fatty acids are used for the biosynthesis of dudomycins: The shorter HMH and the longer HMO. Three HMH residues are found in the smallest compound—dudomycin A, while three HMO residues are used to form the largest compound—dudomycin D. Dudomycins B and C contain both types of hydroxy fatty acids in their structures. The results of gene inactivation studies and sequence analysis demonstrated that only the product of the *dudA* gene is responsible for biosynthesis of dudomycins. The gene encodes an NRPS comprising four domains only: C, A, PCP, and TE. We propose that the A domain activates the l-lysine and loads it on the PCP module. The C domain then attaches two hydroxy fatty acid moieties to the two available amino groups of PCP-bound lysine. The product is released through the hydrolytic activity of the TE domain. We propose that the third hydroxy fatty acid is spontaneously esterified with HMH1 or HMO1, respectively (Figure 2 and Figure 5). It remains unclear if the attachment of the third hydroxy fatty acid occurs before or after the release of the NRPS product. No peaks corresponding to the dudomycin derivatives without HMH3 or HMO3 residue could be identified by HPLC-MS analysis in the extracts of Streptomyces strains harboring the dudomycin biosynthetic cluster.

Attachment of two hydroxy fatty acid residues to a lysine, which is catalyzed by the single-module NRPS encoded by *dudA* is not typical. At least another two cases are known when a single NRPS module performs two rounds of condensation. During the biosynthesis of myxochelins a single-module NRPS encoded by the *mxcG* gene creates the amide linkage between two 2,3-dihydroxybenzoic acid residues and the two amino groups of lysine [24]. During the biosynthesis of vibriobactin the single-module NRPS encoded by the *vibF* gene also catalyzes two condensation events. However, in the case of vibriobactin, two condensation domains are present within *VibF* each of which is likely responsible for one condensation reaction [25].

Three genes, *dudA* to *dudC*, are required for the biosynthesis of dudomycins in *S. albus* Del14 and *S. lividans* Del8. The *dudA* gene encodes the above mentioned NRPS, and genes, *dudB* and *dudC,* encode the putative membrane transporter and transcriptional regulator. No genes involved in precursor supply could be identified in the DNA regions flanking genes, *dudA* to *dudC*, implying that all precursors required for the dudomycin biosynthesis, including the hydroxy fatty acids HMH and HMO are provided by the host metabolism.

**Figure 6:**
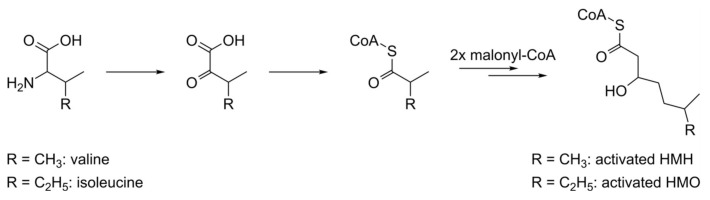
Proposed biosynthesis of HMH and HMO.

*Streptomyces* are known for their extraordinary prevalence of branched-chain fatty acids, which are synthesized by type II fatty acid synthases [26]. Branched-chain amino acids often serve as precursors for biosynthesis of iso and anteiso carboxylic acids. Through oxidation of the amine group, the amino acids are converted into α-keto acids, which are then used as starter units in bacterial fatty acid biosynthesis [27]. Valine and isoleucine with high probability serve as main biosynthetic precursors for HMH and HMO, respectively. These amino acids are converted to 3-methyl-2-oxobutanoic acid and 3-methyl-2-oxopentanoic acid through the action of valine dehydrogenase or homologous enzymes. The formed 2-keto carboxylic acids are decarboxylated and used as starter units by a type II fatty acid synthase which converts them to HMH and HMO after decarboxylative condensation of two malonyl units (Figure 6). The precursor role of l-valine and l-isoleucine in biosynthesis of HMH and HMO is indirectly confirmed by feeding studies [28]. During cultivation in a defined medium with l-valine as nitrogen source, the strain *S. albus* 4E8_del2 produced mostly the smaller dudomycins (dudomycins A and B), which contain mainly HMH, which is derived from l-valine. With l-isoleucine, as a single nitrogen source, the strain produced mostly the bigger dudomycins C and D. These compounds contain mainly HMO, which is derived from l-isoleucine (Figure S25). Furthermore, heavily impaired production of dudomycins was observed when BAC 4E8 was expressed in an *S. albus* delVDH strain [29] with inactivated valine dehydrogenase gene *vdh* (Figure S27). This result suggests that the valine dehydrogenese is involved in the supply of HMH and HMO by oxidation of the branched-chain amino acids l-valine and l-isoleucine. Low dudomycin production by *S. albus* delVDH 4E8_del2 also indicates that other homologs of the valine dehydrogenase with broad substrate specificity are encoded in the genome of *S. albus,* in accordance with previously published data [30].

## 4. Conclusions

In this paper, we report the identification and successful heterologous expression of a new NRPS cluster leading to the production of a group of new compounds called dudomycins A to D. The single-module NRPS which is responsible for dudomycin production activates l-lysine and catalyzes its condensation with two hydroxy fatty acid CoA precursors. Such examples where a single condensation domain catalyzes two condensation steps are very rare. The branched hydroxy fatty acids used for the dudomycin biosynthesis are provided by the host’s metabolism. The amino acids l-valine and l-isoleucine serve as main precursors for their biosynthesis.

## Figures and Tables

**Table 1:** Proposed functions of the genes in the DNA fragment containing the dudomycin gene cluster.

Gene #	Locus Tag	Putative Function
1	SACHL_42600	glycosyltransferase
2	SACHL_42610	hypothetical protein
3	SACHL_42620	phosphatase
4	SACHL_42630	*N,N’*-diacetyllegionaminic acid synthase
5	SACHL_42640	hypothetical protein
6	SACHL_42650	hydrolase
7	SACHL_42660	membrane lipoprotein precursor
8	SACHL_42670	galactose/methyl galactoside import ATP-binding protein
9	SACHL_42680	ribose transport system permease protein
10	SACHL_42690	branched-chain amino acid transport system/permease component
11	SACHL_42700	cytidine deaminase
12	SACHL_42710	pyrimidine-nucleoside phosphorylase
13	SACHL_42720	hypothetical protein
14	SACHL_42730	hypothetical protein
15	SACHL_42740	hypothetical protein
16	SACHL_42750	hypothetical protein
17	SACHL_42760	hypothetical protein
18	SACHL_42770	hypothetical protein
19	SACHL_42780	ubiquinone biosynthesis *O*-methyltransferase
20	SACHL_42790	zinc metallo-peptidase
21	SACHL_42800	hypothetical protein
22 [*dudA*]	SACHL_42810	dimodular nonribosomal peptide synthase
23 [*dudB*]	SACHL_42820	inner membrane transport protein
24 [*dudC*]	SACHL_42830	transcriptional regulator
25	SACHL_42840	demethylrebeccamycin-d-glucose *O*-methyltransferase
26	SACHL_42850	hypothetical protein

## Supplementary Materials

The following are available online at https://www.mdpi.com/2076-2607/8/11/1800/s1. Figure S1: Production of dudomycins by different hosts using different organic solvents for extraction, Figure S2: Mass spectra of dudomycins (**A**–**D**), Figure S3: ^1^HNMR (500 MHz, CDCl_3_) spectrum of dudomycin A, Figure S4: ^1^HNMR (500 MHz, CD_3_OD) spectrum of dudomycin A, Figure S5: 13C NMR (176 MHz, CD_3_OD) spectrum of dudomycin A, Figure S6: DEPT-135 (176 MHz, CD_3_OD) spectrum of dudomycin A, Figure S7, HSQC (700 MHz, CD_3_OD) spectrum of dudomycin A, Figure S8: COSY (700 MHz, CD_3_OD) spectrum of dudomycin A, Figure S9: HMBC (700 MHz, CD_3_OD) spectrum of dudomycin A, Figure S10: (**A**) ^1^HNMR spectrum of dudomycin A showing the irradiation points B (Lys, H-2), C (HMH3, H-3‴), D (HMH2, H-3″) and E (HMH1, H 3′). (**B**–**E**) Selective 1D TOCSY (700 MHz, CD_3_OD) spectra of the corresponding irradiation points, Figure S11: ^1^HNMR (700 MHz, CD_3_OD) spectrum of dudomycin B, Figure S12; DEPT-135 (176 MHz, CD_3_OD) spectrum of dudomycin B showing a 2/1 ratio of the methyl signals of HMH and HMO, Figure S13: HSQC (700 MHz, CD_3_OD) spectrum of dudomycin B, Figure S14: COSY (700 MHz, CD_3_OD) spectrum of dudomycin B, Figure S15: HMBC (700 MHz, CD_3_OD) spectrum of dudomycin B, Figure S16: (**A**) ^1^HNMR spectrum of dudomycin B showing the irradiation points C (H-3″/H-3‴) and C (H-3′). (**B**–**D**) Selective 1D TOCSY (700 MHz, CD_3_OD) spectra of the corresponding irradiation points, Figure S17: H NMR (700 MHz, CD_3_OD) spectrum of dudomycin C, Figure S18: DEPT-135 (176 MHz, CD_3_OD) spectrum of dudomycin C showing an approximate 1/2 ratio of the methyl signals of HMH and HMO, Figure S19: HMBC (700 MHz, CD_3_OD) spectrum of dudomycin C, Figure S20: (**A**) ^1^HNMR spectrum of dudomycin C showing the irradiation points B (H-3″/H-3‴) and C (H-3′). (**B**–**D**) Selective 1D TOCSY (700 MHz, CD_3_OD) spectra of the corresponding irradiation points, Figure S21: ^1^HNMR (700 MHz, CD_3_OD) spectrum of dudomycin D, Figure S22: DEPT-135 (176 MHz, CD_3_OD) spectrum of dudomycin D, Figure S23: HSQC (700 MHz, CD_3_OD) spectrum of dudomycin D, Figure S24: (**A**) ^1^HNMR spectrum of dudomycin D showing the irradiation points B (HMO2/3, H-3″/H-3‴), C (HMO1, H-3′) and D (Lys, H-2). (**B**–**D**) Selective 1D TOCSY (700 MHz, CD_3_OD) spectra of the corresponding irradiation points; Figure S25: Production of dudomycin derivatives in defined medium (DM) with single sources of nitrogen, Figure S26: Production of dudomycins after gene deletion experiments, Figure S27: Decreased production of dudomycin derivatives by *S. albus* delVDH 4E8, Table S1: NMR data of dudomycin A in CD_3_OD, Table S2: NMR data of dudomycin D in CD_3_OD, Table S3: Strains, BACs, plasmids and primers used in this work, Tables S4: Proposed functions of the genes in the DNA fragment containing the dudomycin gene cluster.
