# Supplementary material for: Dudomycins: New Secondary Metabolites Produced after Heterologous Expression of an Nrps Cluster from Streptomyces albus ssp. Chlorinus Nrrl B-24108
Source: Microorganisms. 2020 Nov 16;8(11):1800. doi: 10.3390/microorganisms8111800 (PMC7696016; doi:10.3390/microorganisms8111800)
Supplement: Supplementary file 1 [file microorganisms-08-01800-s001.pdf]

*microorganisms*

*Article*

# Dudomycins: new secondary metabolites produced after heterologous expression of an NRPS cluster from *Streptomyces albus* ssp. *chlorinus* NRRL B-24108

**Constanze Lasch [1], Marc Stierhof [1], Marta Rodríguez Estévez [1], Maksym Myronovskyi [1], Josef Zapp [2] and Andriy Luzhetskyy [1,3,*]**

[1] Pharmaceutical Biotechnology, Saarland University, Saarbruecken, Germany; constanze.lasch@uni-saarland.de (C.L.), m.stierhof@t-online.de (M.S.), marta.rodriguezestevez@uni-saarland.de (M.R.), maksym.myronovskyi@uni-saarland.de (M.M.), a.luzhetskyy@mx.uni-saarland.de (A.L.)

[2] Pharmaceutical Biology, Saarland University, Saarbruecken, Germany; j.zapp@mx.uni-saarland.de (J.Z.)

[3] Helmholtz Institute for Pharmaceutical Research Saarland, Saarbruecken, Germany

* Correspondence: a.luzhetskyy@mx.uni-saarland.de; +49 681 302 70200 (A.L.)

Received: date; Accepted: date; Published: date

**Supplementary.**

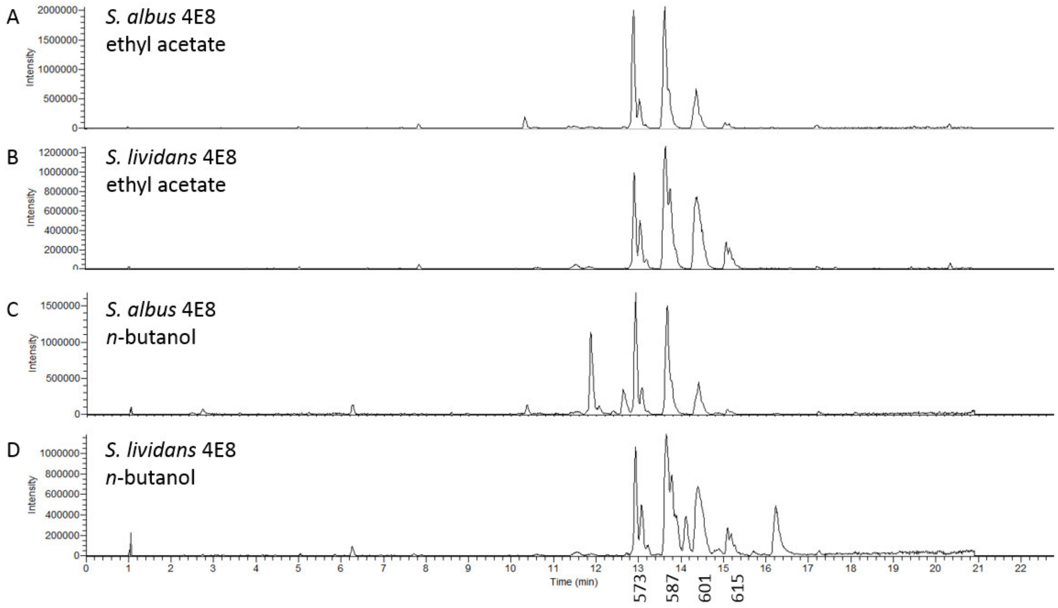


**Figure S1.** Production of dudomycins by different hosts using different organic solvents for extraction. Base Peak Chromatogram (BPC) extracted for masses M+H+ [573-574], [587-588], [601-602], [615-616]. Butanolic extractions led to more background peaks in crude extracts. *S. albus* 4E8 showed higher production levels compared to *S. lividans* 4E8.

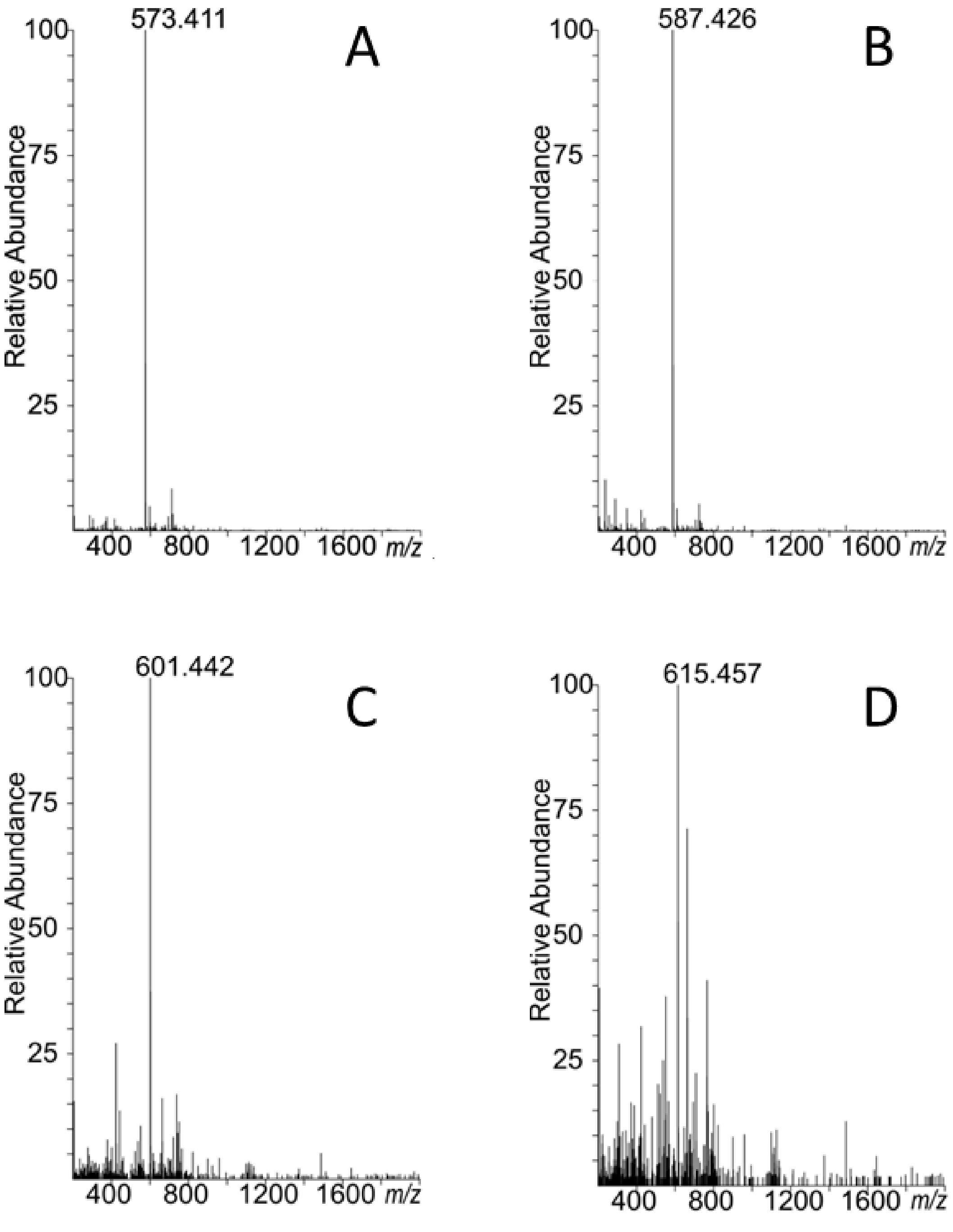


**Figure S2.** A to D: Mass spectra of dudomycins A to D.

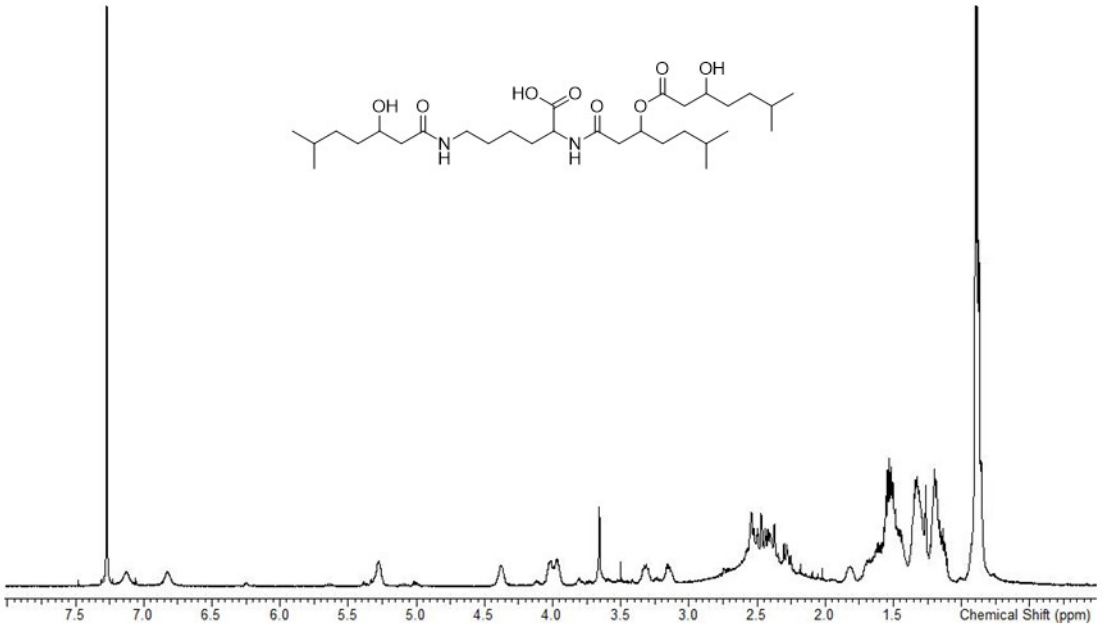


**Figure S3.** $^1$H NMR (500 MHz, $CDCl_3$) spectrum of dudomycin A.

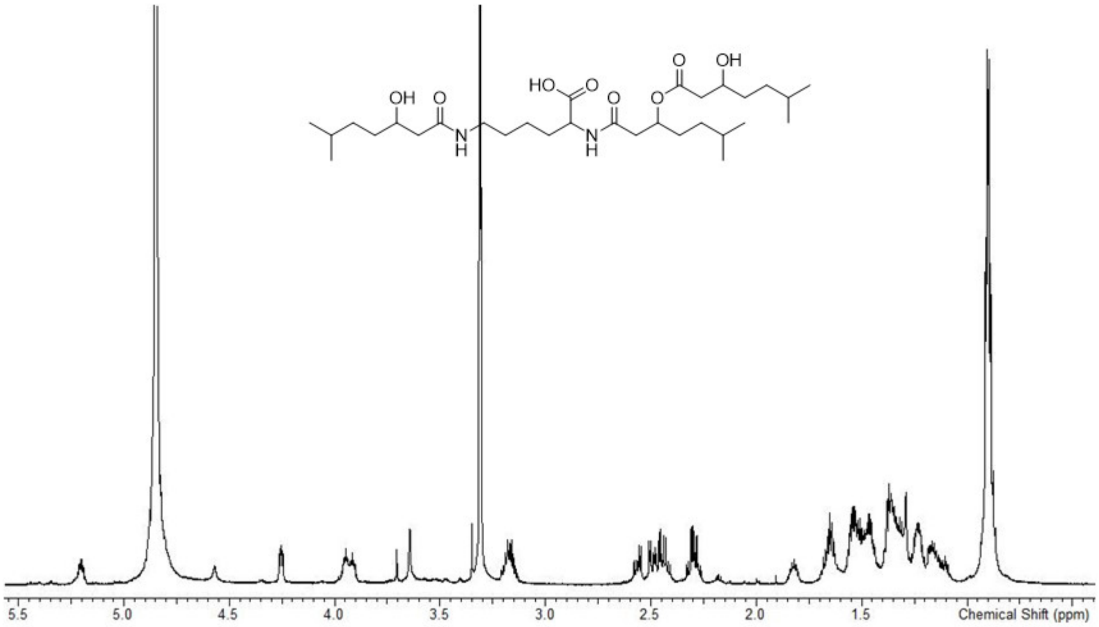


**Figure S4.** $^1$H NMR (500 MHz, $CD_3OD$) spectrum of dudomycin A.

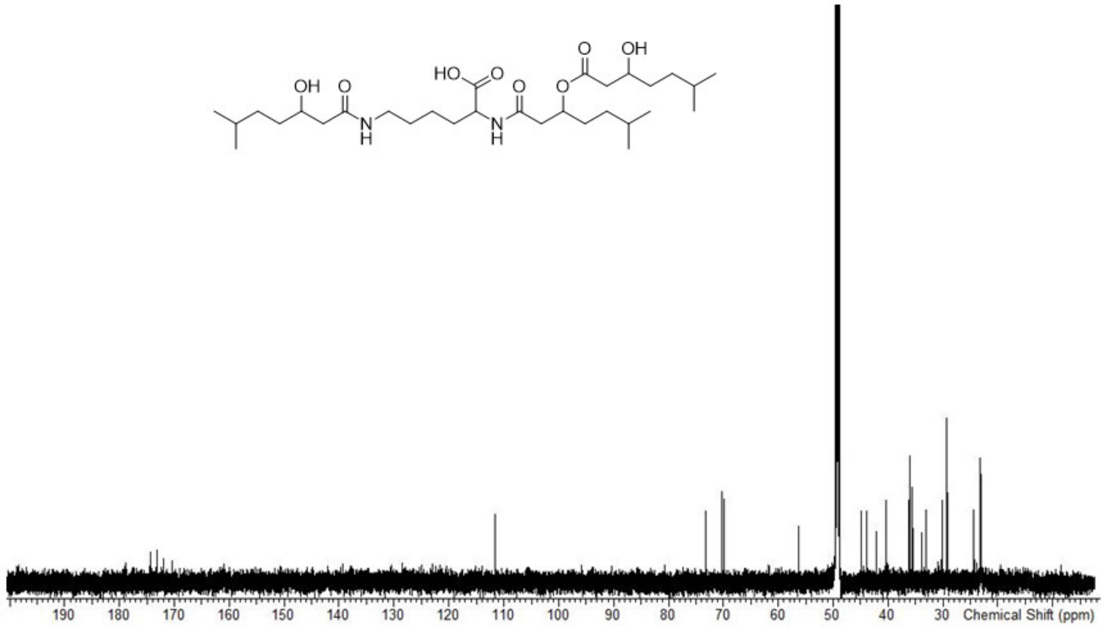


**Figure S5.** $^{13}C$ NMR (176 MHz, $CD_3OD$) spectrum of dudomycin A.

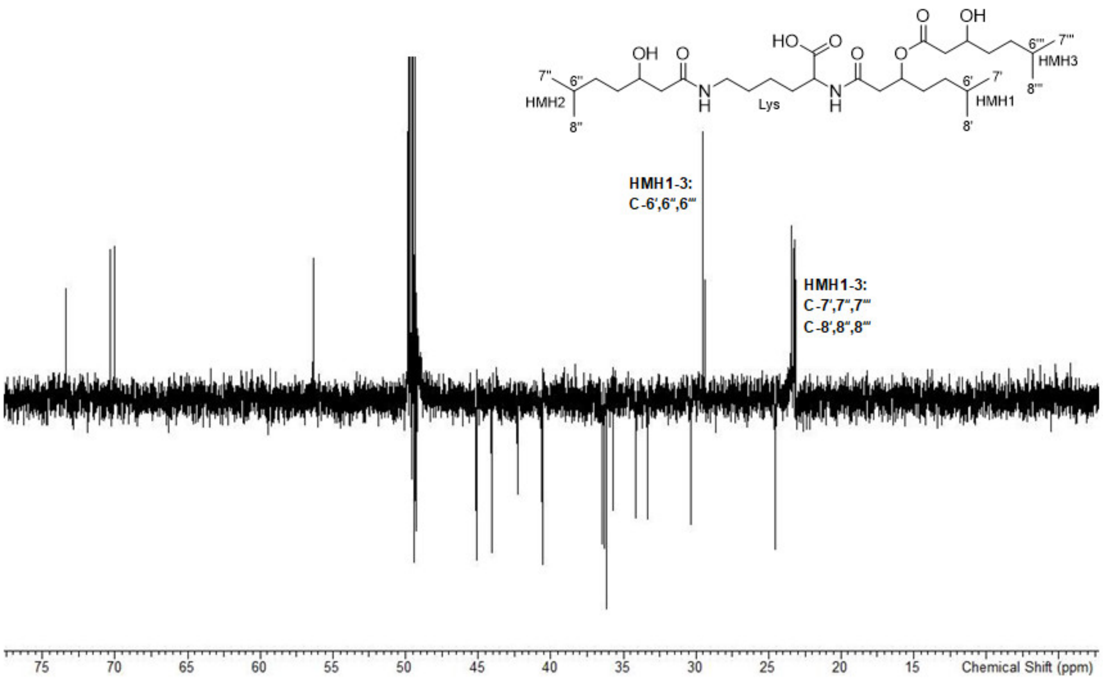


**Figure S6**. DEPT-135 (176 MHz, $CD_3OD$) spectrum of dudomycin A.

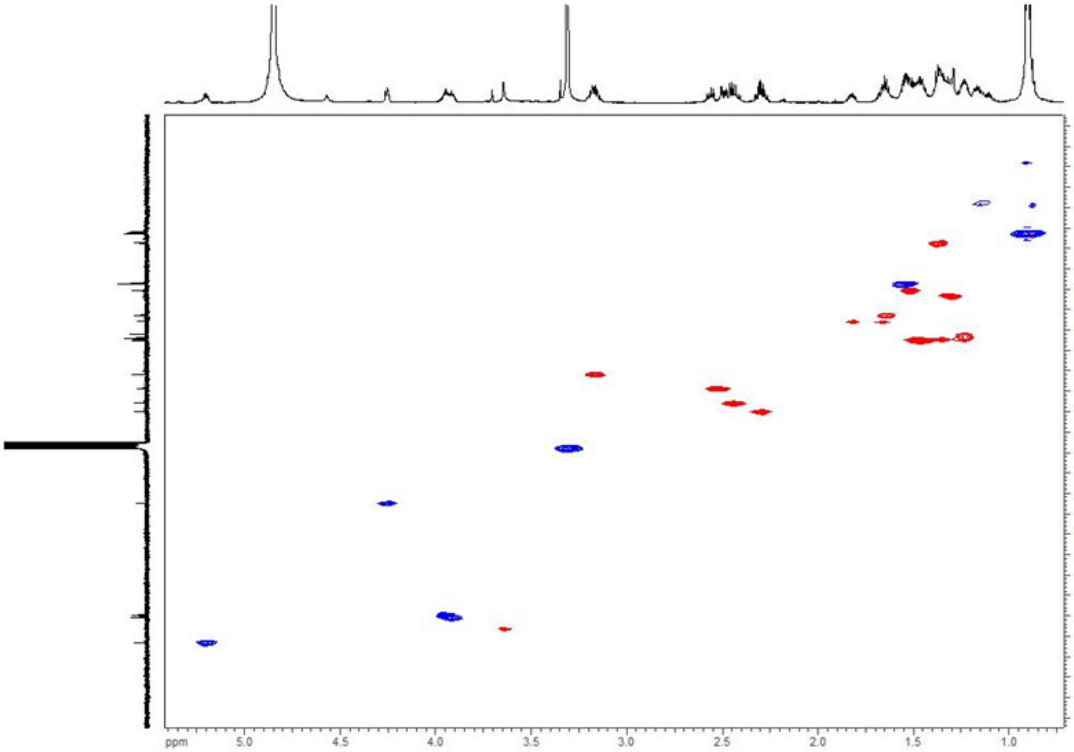


**Figure S7.** HSQC (700 MHz, $CD_3OD$) spectrum of dudomycin A.

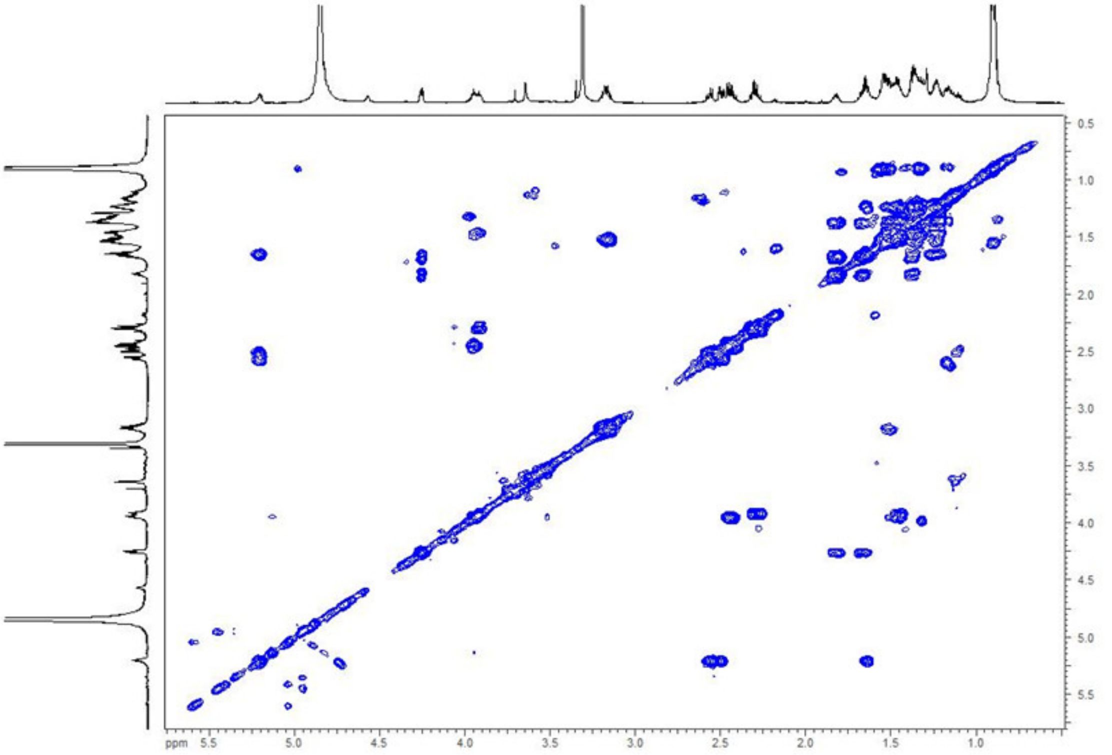


**Figure S8.** COSY (700 MHz, $CD_3OD$) spectrum of dudomycin A.

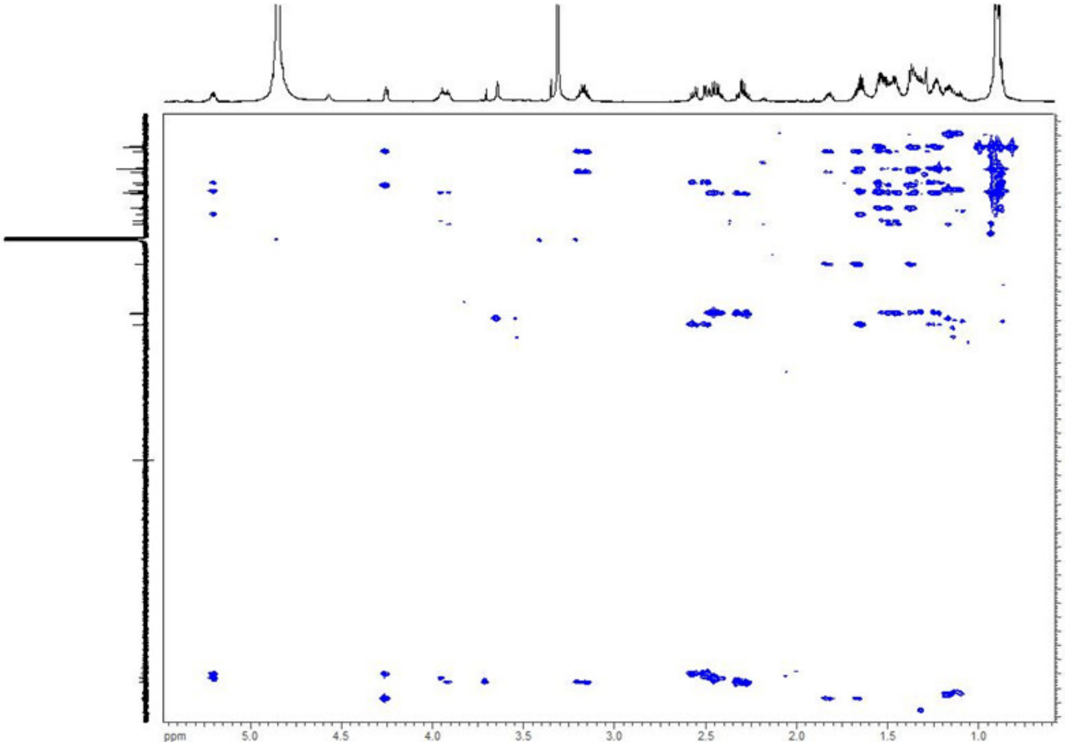


**Figure S9.** HMBC (700 MHz, $CD_3OD$) spectrum of dudomycin A.

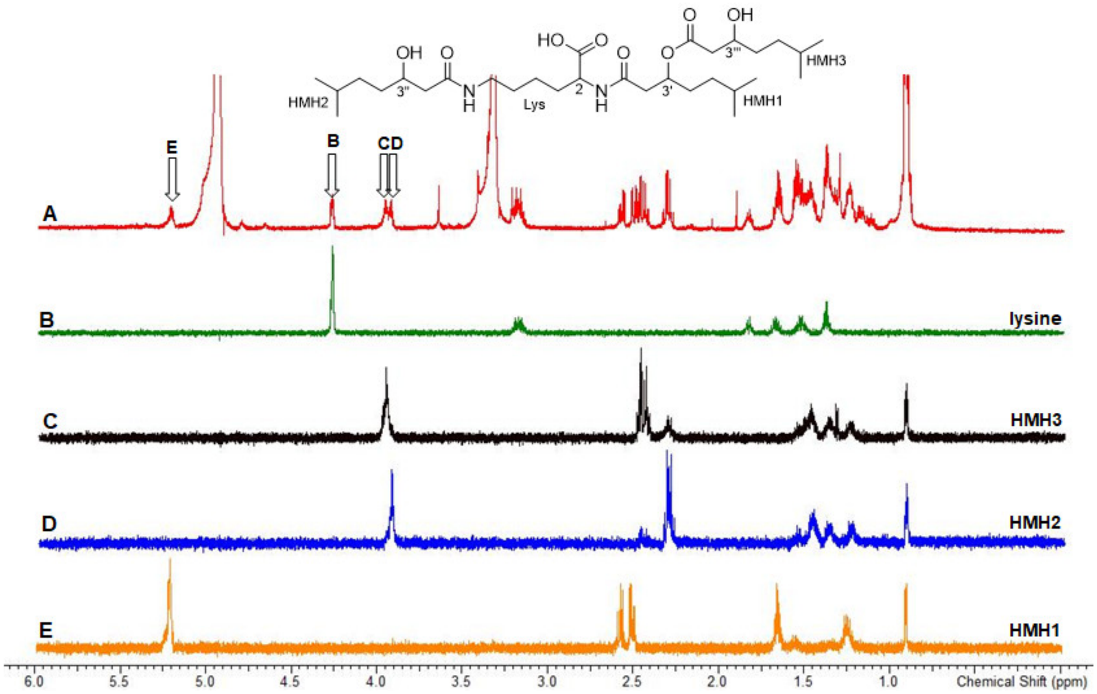


**Figure S10.** A. $^{1}H$ NMR spectrum of dudomycin A showing the irradiation points B (Lys, H-2), C (HMH3, H-3′′′), D (HMH2, H-3′′) and E (HMH1, H 3′). B-E. Selective 1D TOCSY (700 MHz, $CD_3OD$) spectra of the corresponding irradiation points.

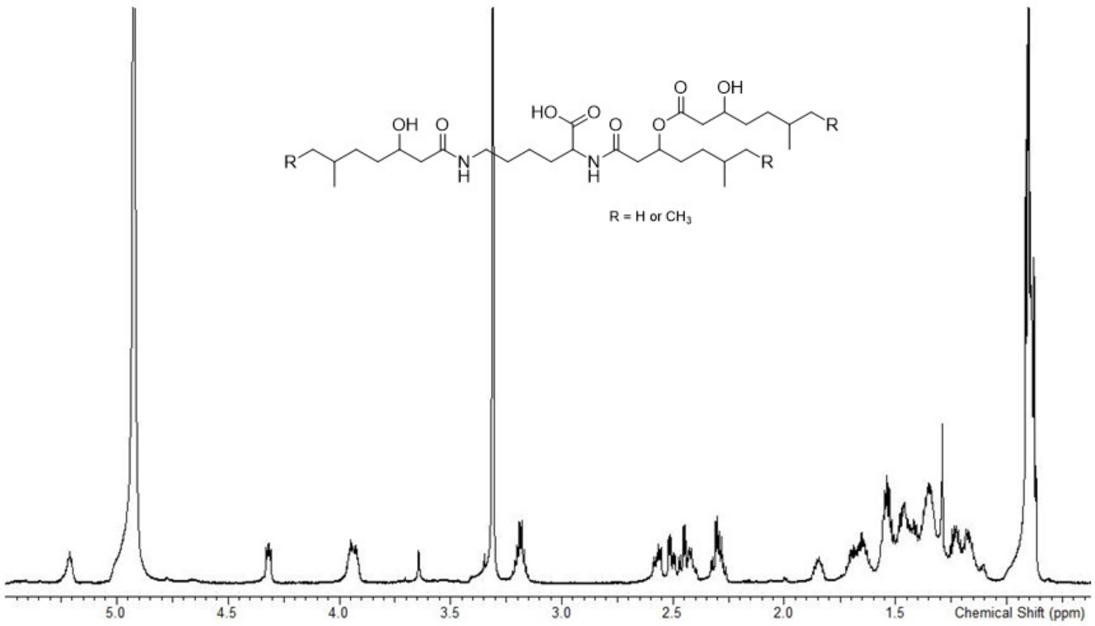


**Figure S11.** $^{1}H$ NMR (700 MHz, $CD_3OD$) spectrum of dudomycin B.

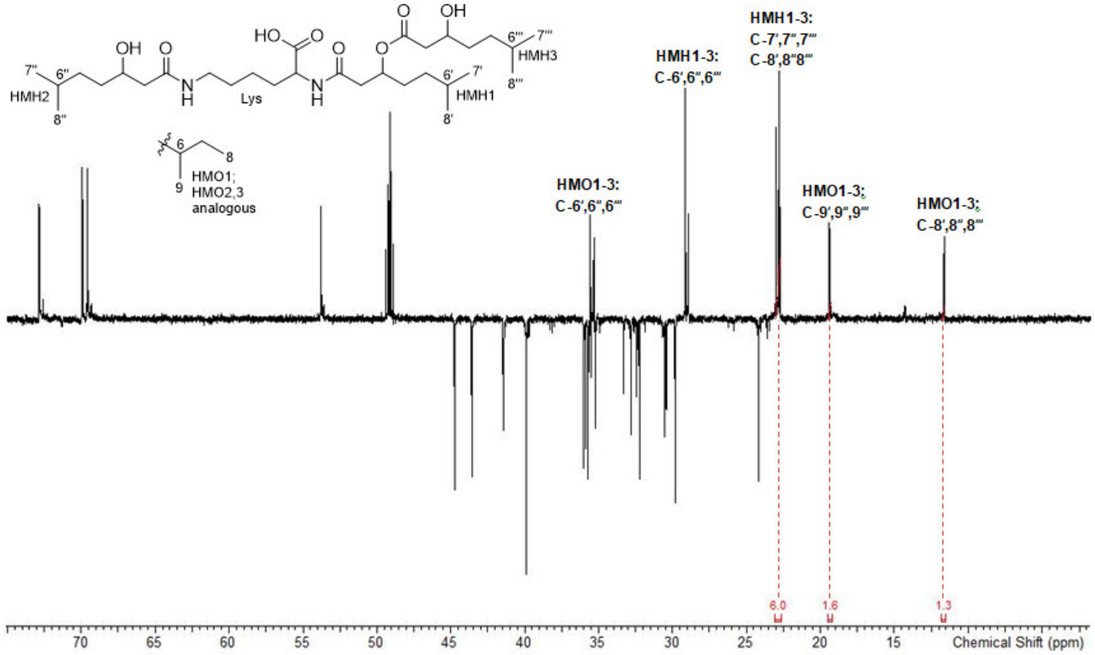


**Figure S12.** DEPT-135 (176 MHz, CD3OD) spectrum of dudomycin B showing a 2/1 ratio of the methyl signals of HMH and HMO.

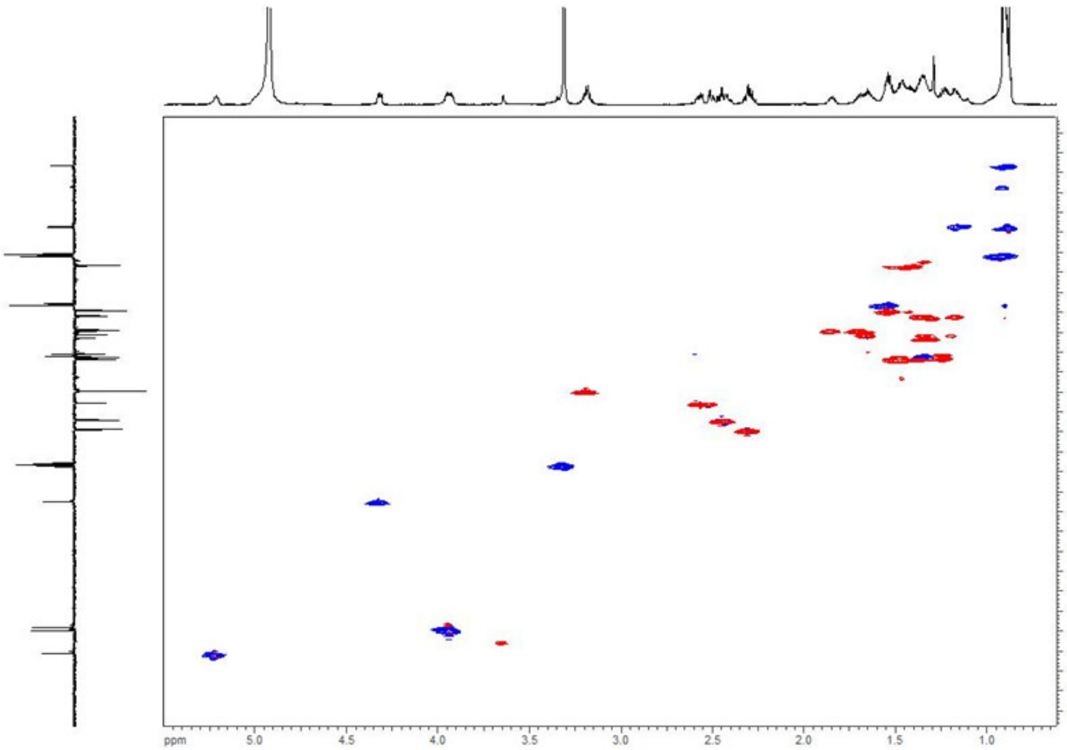


**Figure S13.** HSQC (700 MHz, $CD_3OD$) spectrum of dudomycin B.

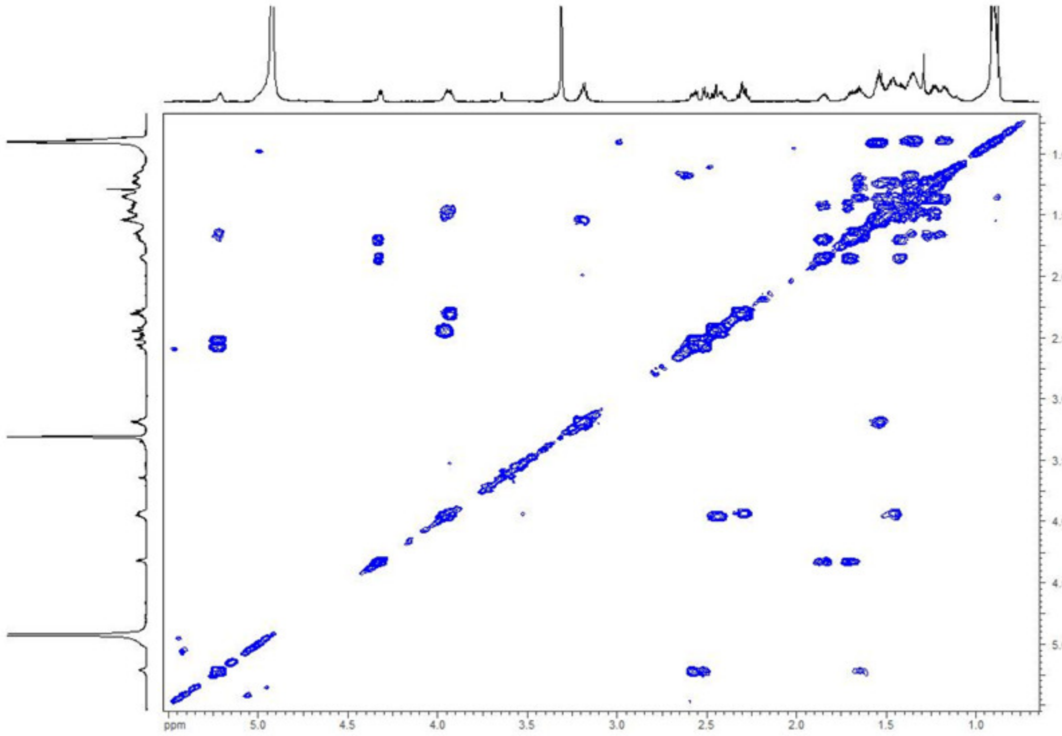


**Figure S14.** COSY (700 MHz, $CD_3OD$) spectrum of dudomycin B.

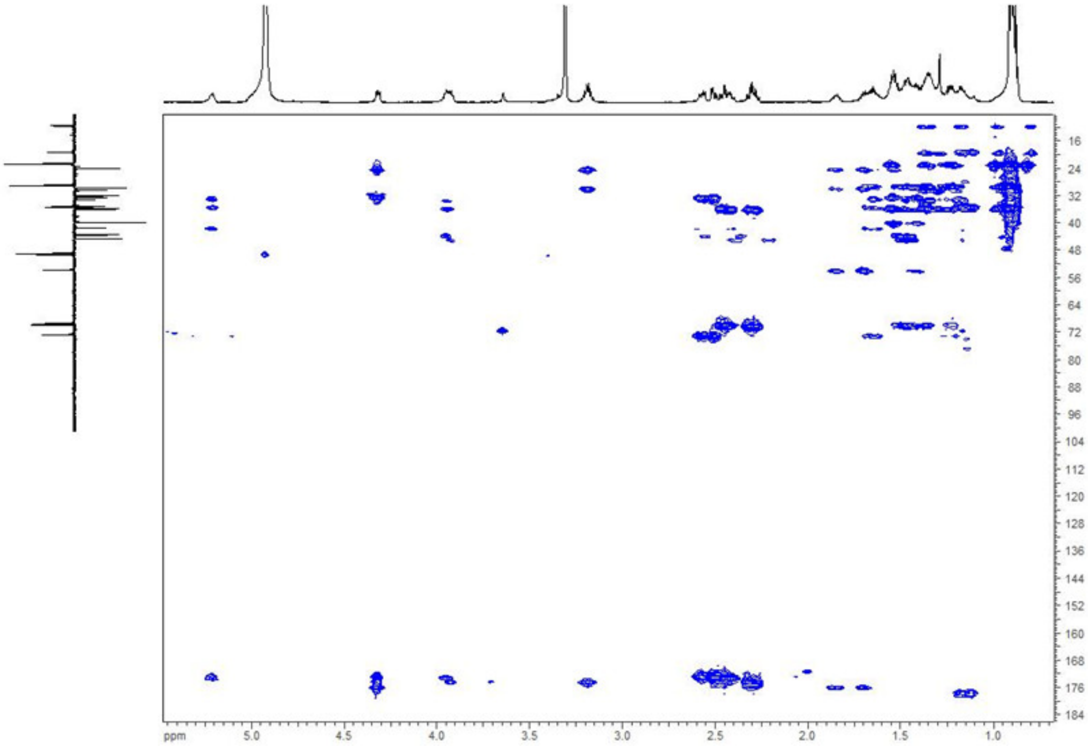


**Figure S15.** HMBC (700 MHz, $CD_3OD$) spectrum of dudomycin B.

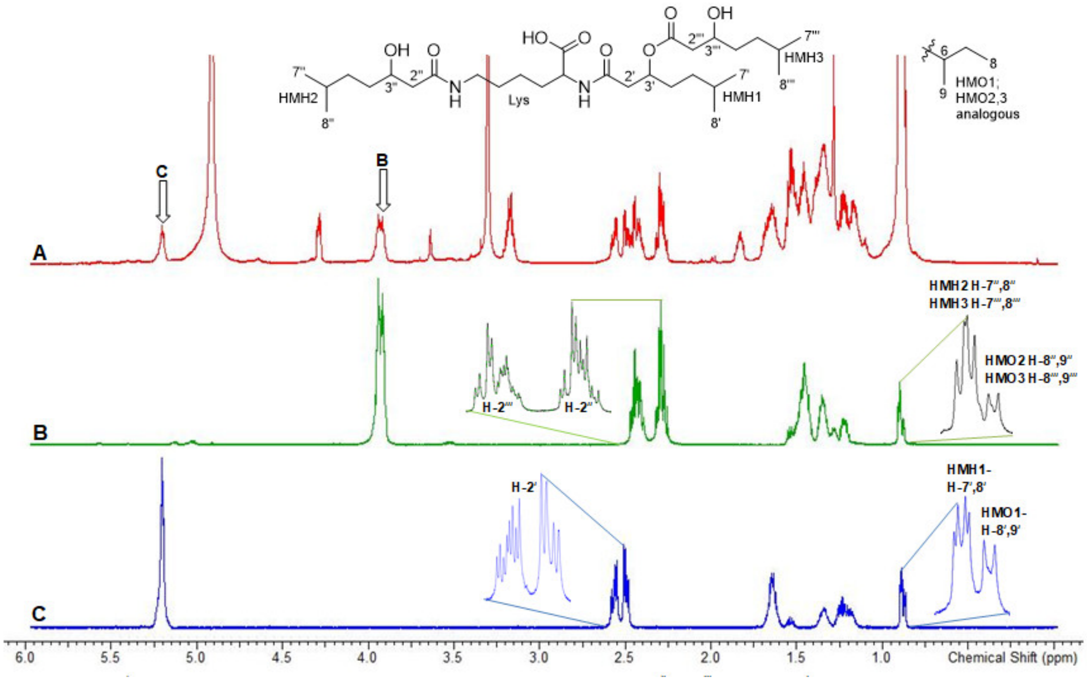


**Figure S16.** A. $^1$H NMR spectrum of dudomycin B showing the irradiation points B (H-3′′/ H-3′′′) and C (H-3′). B-D. Selective 1D TOCSY (700 MHz, $CD_3OD$) spectra of the corresponding irradiation points.

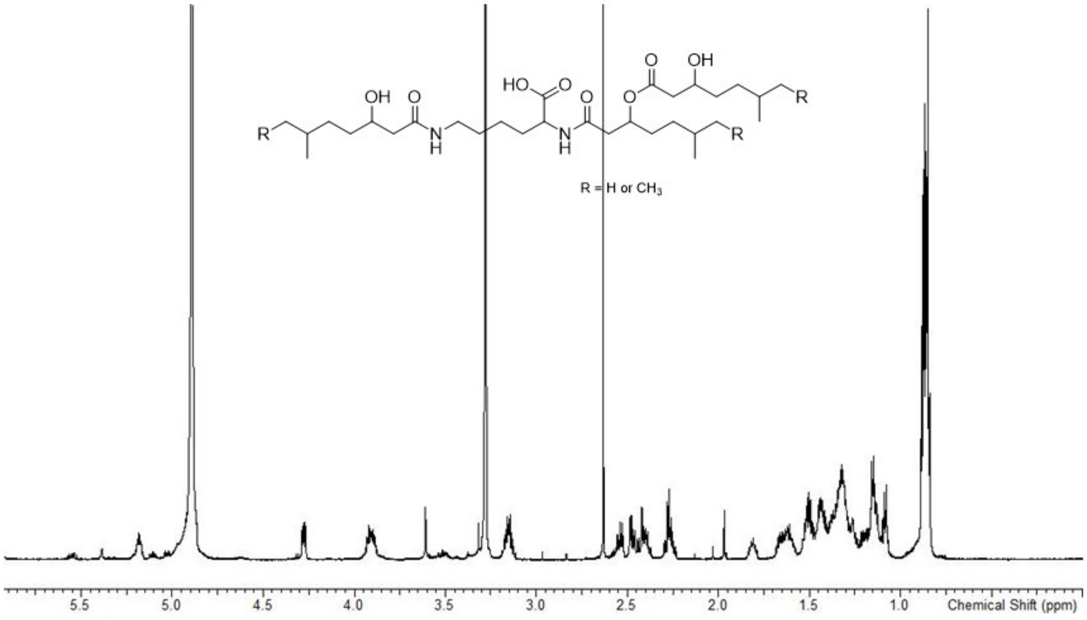


**Figure S17.** $^1$H NMR (700 MHz, $CD_3OD$) spectrum of dudomycin C.

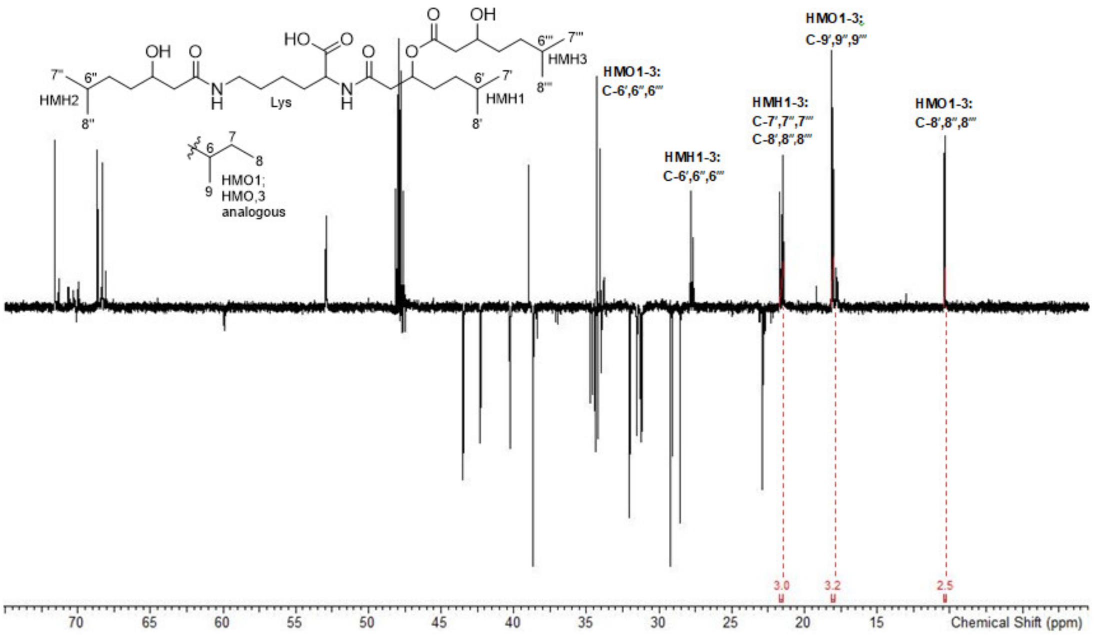


**Figure S18.** DEPT-135 (176 MHz, $CD_3OD$) spectrum of dudomycin C showing an approximate 1/2 ratio of the methyl signals of HMH and HMO.

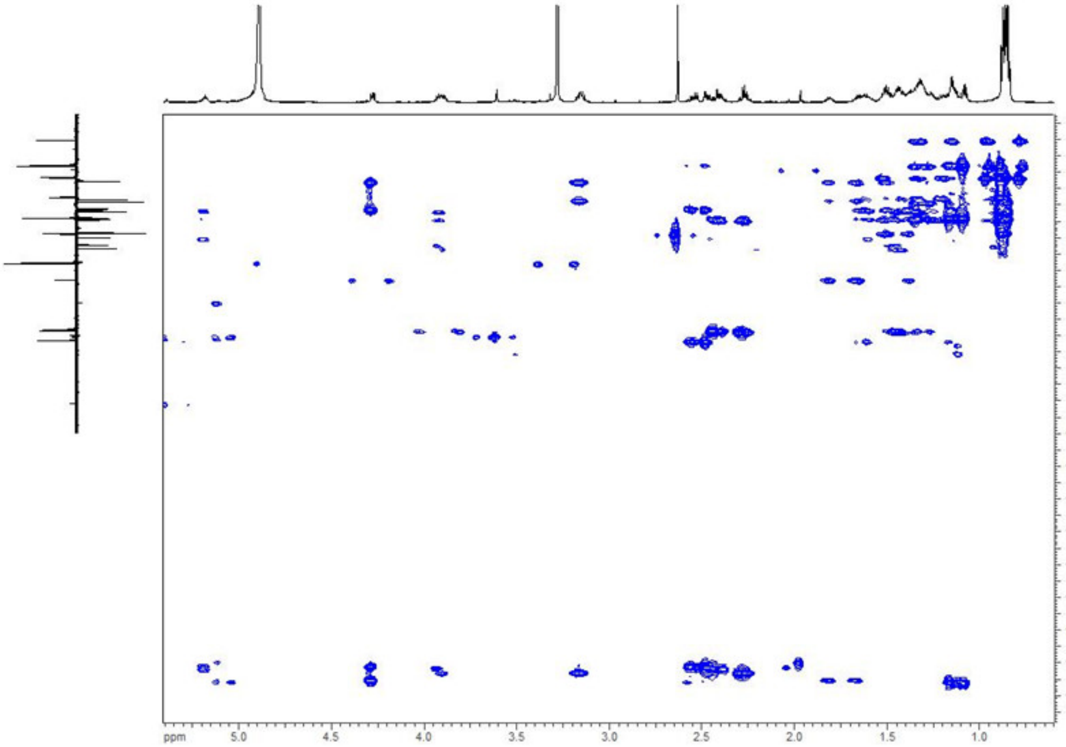


**Figure S19.** HMBC (700 MHz, $CD_3OD$) spectrum of dudomycin C.

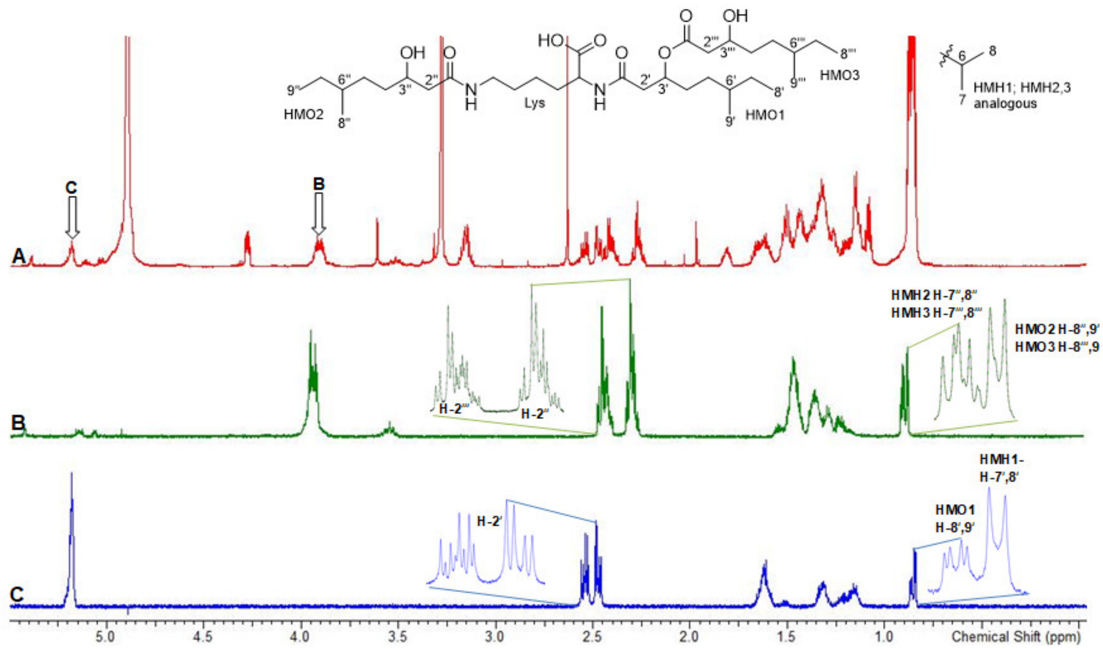


**Figure S20.** A. $^{1}$H NMR spectrum of dudomycin C showing the irradiation points B (H-3′′/ H-3′′′) and C (H-3′). B-D. Selective 1D TOCSY (700 MHz, $CD_3OD$) spectra of the corresponding irradiation points.

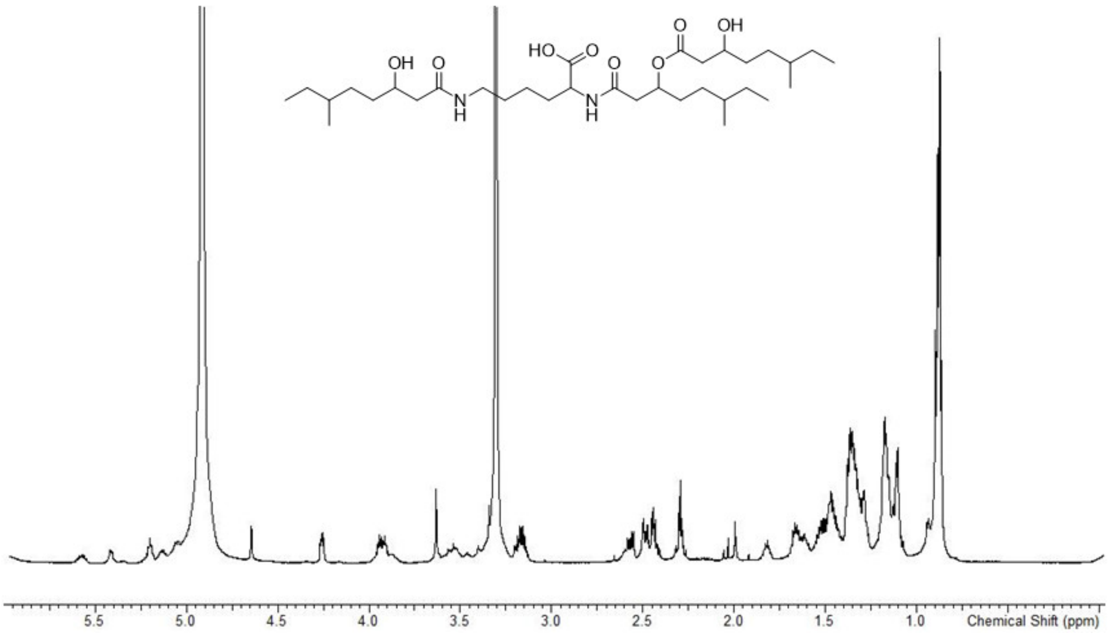


**Figure S21.** $^{1}H$ NMR (700 MHz, $CD_3OD$) spectrum of dudomycin D.

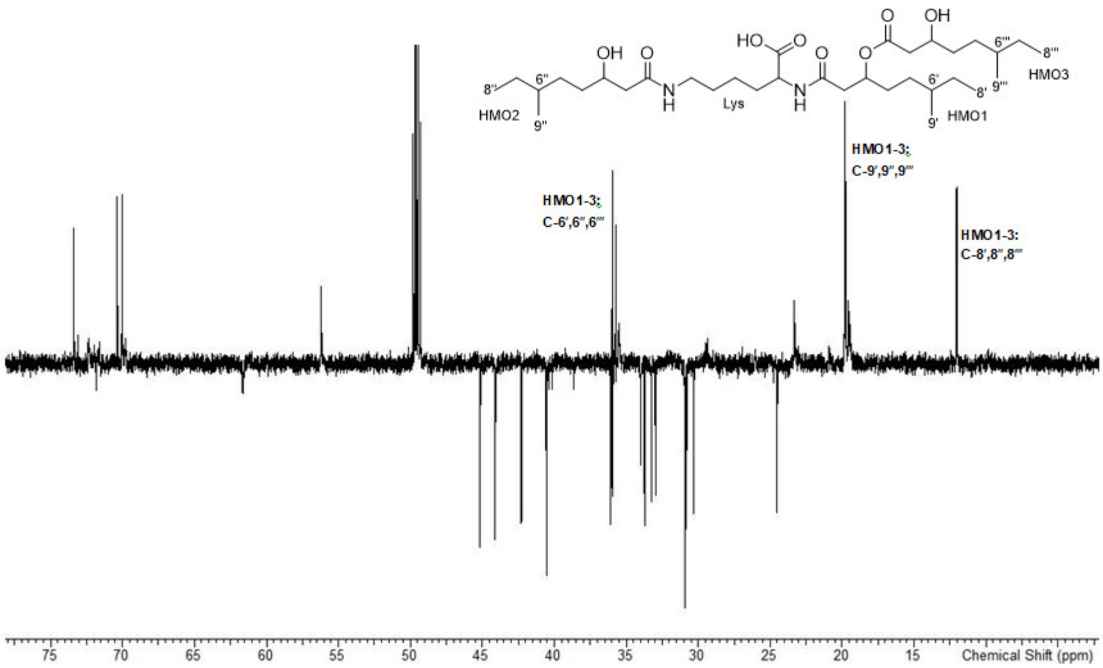


**Figure S22.** DEPT-135 (176 MHz, $CD_3OD$) spectrum of dudomycin D.

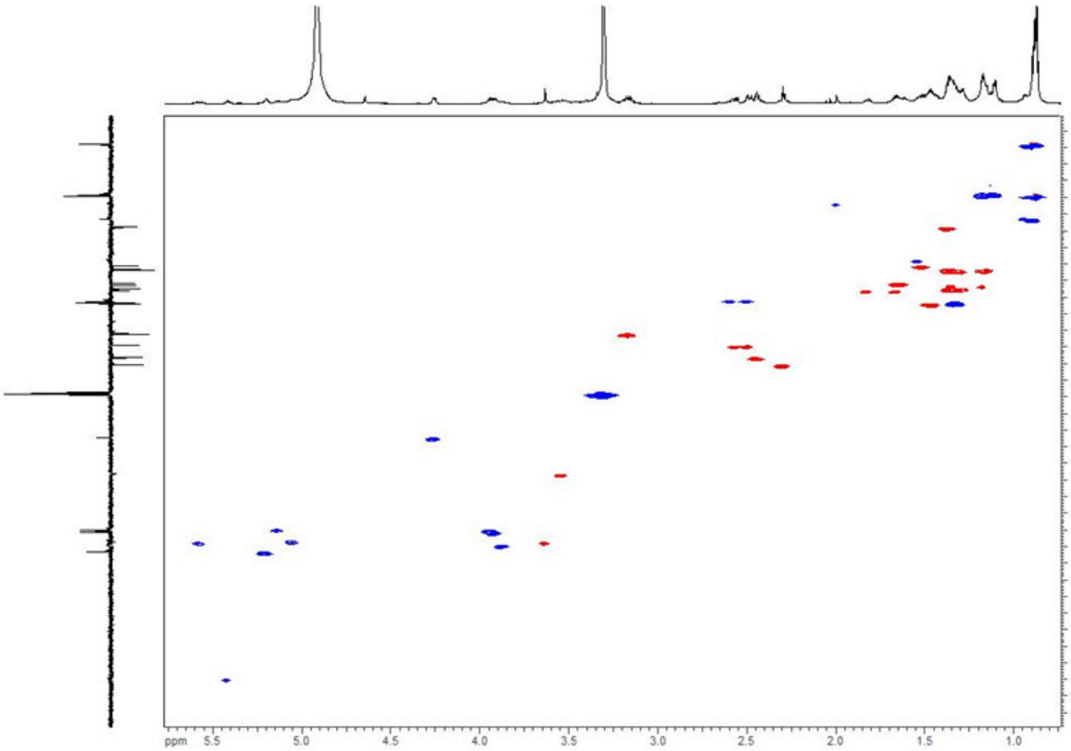


**Figure S23.** HSQC (700 MHz, $CD_3OD$) spectrum of dudomycin D.

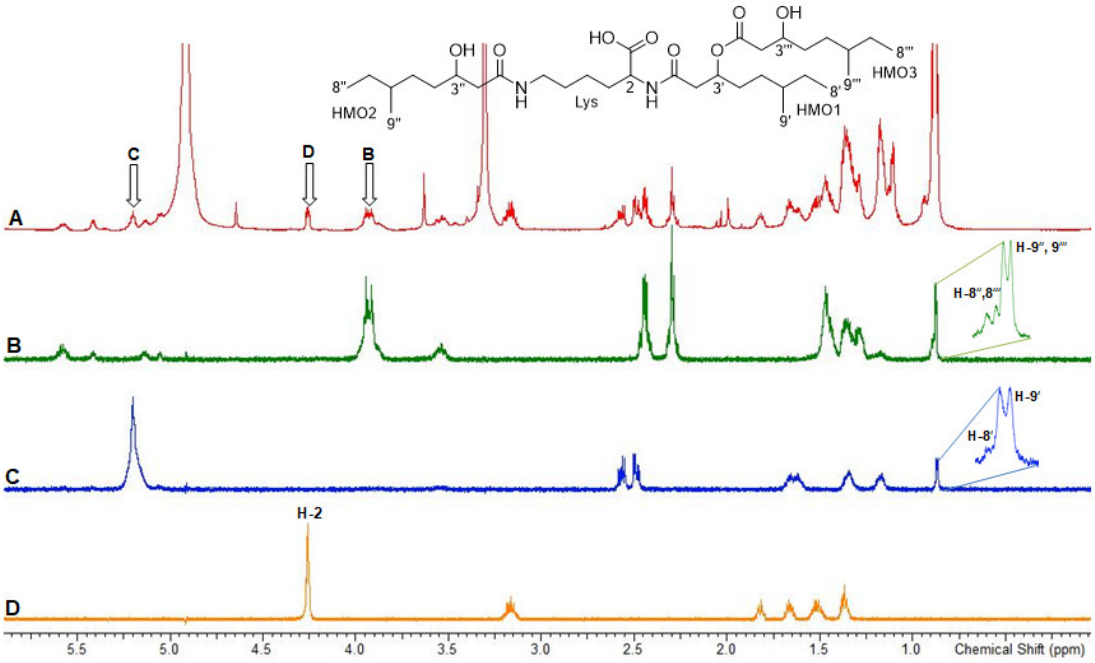


**Figure S24.** A. $^{1}$H NMR spectrum of dudomycin D showing the irradiation points B (HMO2/3, H-3′′/ H-3′′′), C (HMO1, H-3′) and D (Lys, H-2). B D. Selective 1D TOCSY (700 MHz, $CD_3OD$) spectra of the corresponding irradiation points.

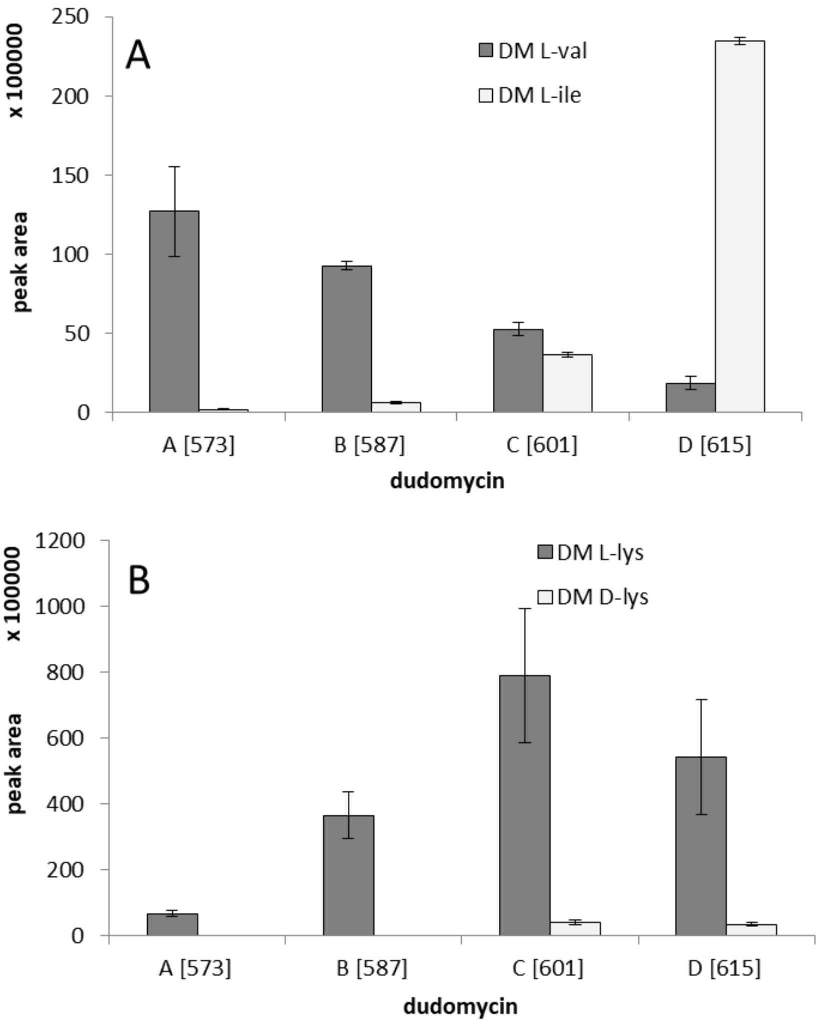


**Figure S25.** Production of dudomycin derivatives in defined medium (DM) with single sources of nitrogen.

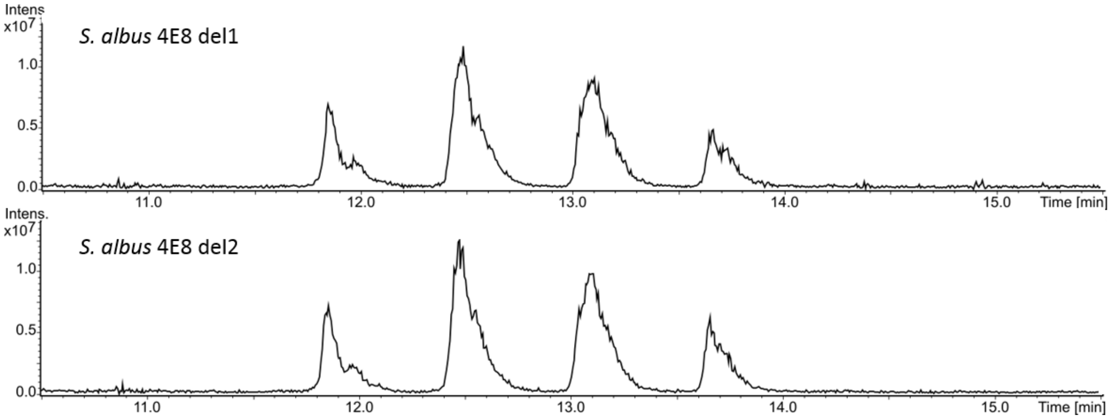


**Figure S26.** Production of dudomycins after gene deletion experiments. BPC extracted for masses [573-574], [587-588], [601-602], [615-616].

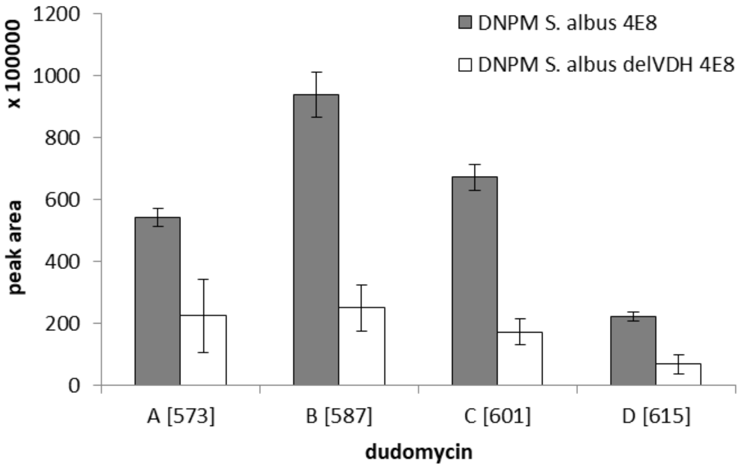


**Figure S27.** Decreased production of dudomycin derivatives by *S. albus* delVDH 4E8.

**Table S1.** NMR data of dudomycin A in $CD_3OD$.

| unit | no. | $\delta_C$, type | δH, (J in Hz) |
|---|---|---|---|
| Lys | 1 | 178.9, C | - |
| | 2 | 56.2, CH | 4.26, dd (4.8, 7.5) |
| | 3 | 34.0, $CH_2$ | 1.83, m; 1.677, m |
| | 4 | 24.3, $CH_2$ | 1.38, m |
| | 5 | 30.2, $CH_2$ | 1.51, m |
| | 6 | 40.4, $CH_2$ | 3.17, m |
| | NH-1 | - | 7.12, bs[1] |
| | NH-2 | - | 6.81, bs[1] |
| | | | |
| HMH1 | 1' | 171.9, C | - |
| | 2' | 42.2, $CH_2$ | 2.56, dd (7.7, 14.1); 2.50, dd (5.1, 14.4) |
| | 3' | 73.3, CH | 5.20, dd (4.94, 7.5) |
| | 4' | 33.2, $CH_2$ | 1.64, m |
| | 5' | 35.6, $CH_2$ | 1.23, m |
| | 6' | 29.2, CH | 1.54, m |
| | 7' | 23.1, $CH_3$ | 0.88, d (6.5) |
| | 8' | 23.0, $CH_3$ | 0.89, d (6.5) |
| | | | |
| HMH2 | 1'' | 174.3 | - |
| | 2'' | 44.9, $CH_2$ | 2.32, dd (4.5, 13.9); 2.28, dd (8.0, 13.9) |
| | 3'' | 70.2, CH | 3.92, m |
| | 4'' | 36.3, $CH_2$ | 1.47, m |
| | 5'' | 36.0, $CH_2$ | 1.23, m |
| | 6'' | 29.4, CH | 1.56, m |
| | 7'' | 23.1, $CH_3$ | 0.90, d (4.4) |
| | 8'' | 23.1, $CH_3$ | 0.89, d (4.4) |
| | | | |
| HMH3 | 1''' | 173.2 | - |
| | 2''' | 43.9, $CH_2$ | 2.47, dd (5.4, 14.8); 2.42, d (7.6, 14.9) |
| | 3''' | 69.9, CH | 3.95, m |
| | 4''' | 36.2, $CH_2$ | 1.49, m; 1.348, m |
| | 5''' | 36.0, $CH_2$ | 1.23, m |
| | 6''' | 29.4, CH | 1.56, m |
| | 7''' | 23.3, $CH_3$ | 0.91, d (4.8) |
| | 8''' | 23.3, $CH_3$ | 0.90, d (4.8) |

[1]NH Signals measured in $CDCl_3$

**Table S2.** NMR data of dudomycin D in $CD_3OD$.

| unit | no. | δC‡, type | δH, (J in Hz) |
|---|---|---|---|
| Lys | 2 | 55.8, CH | 4.26, dd (4.5, 6.8) |
| | 3 | 33.6, $CH_2$ | 1.82, m; 1.66, m |
| | 4 | 24.1, $CH_2$ | 1.38, m |
| | 5 | 29.9, $CH_2$ | 1.51, m |
| | 6 | 40.1, $CH_2$ | 3.17, m |
| HMO1 | 2' | 41.9, $CH_2$ | 2.57, dd (8.5, 14.4); 2.49, dd (4.9, 14.4) |
| | 3' | 72.9, CH | 5.20, m |
| | 4' | 32.8, $CH_2$ | 1.64, m |
| | 5' | 32.5, $CH_2$ | 1.36, m; 1.18, m |
| | 6' | 35.3, CH | 1.34, m |
| | 7' | 30.4, $CH_2$ | 1.33, m; 1,17, m |
| | 8' | 11.6, $CH_3$ | 0.88, ovl |
| | 9' | 19.3, $CH_3$ | 0.87, d (6.0) |
| HMO2 | 2'' | 44.7, $CH_2$ | 2.31, dd (4.9, 13.5); 2.28, dd (8.2, 13.6) |
| | 3'' | 69.9, CH | 3.92, m |
| | 4'' | 35.6, $CH_2$ | 1.47, m |
| | 5'' | 33.3, $CH_2$ | 1.30, m; 1.37, m |
| | 6'' | 35.5, CH | 1.34, m |
| | 7'' | 30.5, $CH_2$ | 1.35, m; 1.37, m |
| | 8'' | 11.6, $CH_3$ | 0.89, ovl |
| | 9'' | 19.4, $CH_3$ | 0.88, d (5.4) |
| HMO3 | 2''' | 43.7, $CH_2$ | 2.46, dd (5.5, 15.1); 2.424, d (8.2, 15.1) |
| | 3''' | 69.6, CH | 3.94, m |
| | 4''' | 35.5, $CH_2$ | 1.47, m |
| | 5''' | 33.3, $CH_2$ | 1.30, m; 1.37, m |
| | 6''' | 35.5, CH | 1.34, m |
| | 7''' | 30.5, $CH_2$ | 1.35, m; 1.37, m |
| | 8''' | 11.6 $CH_3$ | 0.89, ovl |
| | 9''' | 19.4, $CH_3$ | 0.88, d (5.4) |

‡from DEPT-135 measurement

**Table S3.** Strains, BACs, plasmids and primers used in this work.

| **Material** | **Purpose** |
|---|---|
| **A. Bacterial strains** | |
| *Streptomyces albus* Del14 | optimized heterologous host [1] |
| *Streptomyces lividans* Del8 | optimized heterologous host [2] |
| *Streptomyces albus* J1074 delVDH | heterologous host [3] |
| *Escherichia coli* GB05 RedCC | cloning host [Helmholtz-Institut für Pharmazeutische Forschung Saarland (HIPS)] |
| *Escherichia coli* ET12567 pUB307 | alternate host intergeneric conjugation [4] |
| **B. BACs** | |
| 4E8 | heterologous expression of NRPS cluster |
| 4E8 del1 | determination left border of NRPS cluster |
| 4E8 del2 | determination right border of NRPS cluster |
| **C. Plasmids** | |
| pUC19 | ampicillin resistance marker |
| pXCM hygformax | hygromycin resistance marker |
| **D. PCR primer** | |
| 20190429_1_fw [4E8 del1] | CAGGGAGGAGGCCACCCCGCGCCGGTAGAAGACGCCCAGCAGGCCGACCGCG TCAGGTGGCACTTTTCG |
| 20190429_1_rev [4E8 del1] | GATCGCGTGCATGCCCAGATGGTCGCGCGCATACCGCCGTCGGAACGGGCGCG GATATCCTACTATGCCGAGGTATAATGTAGCCAGCGTGTTACCAATGCTTAATC AGTG |
| 20190429_2_fw [4E8 del2] | GGCGACGGCTCCCCGGGCCCGCGAGCCCACGACTCCCGTCCCCACGGCCATCA GGCGCCGGGGGCGGTGT |
| 20190429_2_rev [4E8 del2] | GCGATGAGTATCCGTACTCATGTCCGGGCCGCGGGTGCCGCCTAGCGTGAAAT ACTTGACATATCACTGT |

Table S4. Proposed functions of the genes in the DNA fragment containing the dudomycin gene cluster

| **gene #** | **locus tag** | **putative function** |
|---|---|---|
| 1 | SACHL_42600 | glycosyltransferase |
| 2 | SACHL_42610 | hypothetical protein |
| 3 | SACHL_42620 | phosphatase |
| 4 | SACHL_42630 | N,N'-diacetyllegionaminic acid synthase |
| 5 | SACHL_42640 | hypothetical protein |
| 6 | SACHL_42650 | hydrolase |
| 7 | SACHL_42660 | membrane lipoprotein precursor |
| 8 | SACHL_42670 | galactose/methyl galactoside import ATP-binding protein |
| 9 | SACHL_42680 | ribose transport system permease protein |
| 10 | SACHL_42690 | branched-chain amino acid transport system / permease component |
| 11 | SACHL_42700 | cytidine deaminase |
| 12 | SACHL_42710 | pyrimidine-nucleoside phosphorylase |
| 13 | SACHL_42720 | hypothetical protein |
| 14 | SACHL_42730 | hypothetical protein |
| 15 | SACHL_42740 | hypothetical protein |
| 16 | SACHL_42750 | hypothetical protein |
| 17 | SACHL_42760 | hypothetical protein |
| 18 | SACHL_42770 | hypothetical protein |
| 19 | SACHL_42780 | ubiquinone biosynthesis O-methyltransferase |
| 20 | SACHL_42790 | zinc metallo-peptidase |
| 21 | SACHL_42800 | hypothetical protein |
| 22 [*dudA*] | SACHL_42810 | dimodular nonribosomal peptide synthase |
| 23 [*dudB*] | SACHL_42820 | inner membrane transport protein |
| 24 [*dudC*] | SACHL_42830 | transcriptional regulator |
| 25 | SACHL_42840 | demethylrebeccamycin-D-glucose O-methyltransferase |
| 26 | SACHL_42850 | hypothetical protein |

REFERENCES

[1] M. Myronovskyi, B. Rosenkränzer, S. Nadmid, P. Pujic, P. Normand, and A. Luzhetskyy, “Generation of a cluster-free Streptomyces albus chassis strains for improved heterologous expression of secondary metabolite clusters,” *Metab. Eng.*, vol. 49, pp. 316–324, Sep. 2018, doi: 10.1016/j.ymben.2018.09.004.

[2] Y. Ahmed, Y. Rebets, M. R. Estévez, J. Zapp, M. Myronovskyi, and A. Luzhetskyy, “Engineering of Streptomyces lividans for heterologous expression of secondary metabolite gene clusters,” *Microb. Cell Fact.*, vol. 19, no. 1, p. 5, Dec. 2020, doi: 10.1186/s12934-020-1277-8.

[3] N. Manderscheid, “Strain development for heterologous expression of secondary metabolite clusters in actinobacteria,” Universität des Saarlandes, 2015.

[4] F. Flett, V. Mersinias, and C. P. Smith, “High efficiency intergeneric conjugal transfer of plasmid DNA from Escherichia coli to methyl DNA-restricting streptomycetes,” *FEMS Microbiol. Lett.*, vol. 155, no. 2, pp. 223–229, Jan. 2006, doi: 10.1111/j.1574-6968.1997.tb13882.x.
